# Multi-omics analysis untangles the crosstalk between intratumor microbiome, lactic acid metabolism and immune status in lung squamous cell carcinoma

**DOI:** 10.3389/fimmu.2025.1603822

**Published:** 2025-06-11

**Authors:** Xun Qiu, Dan Li

**Affiliations:** Department of Medical Oncology, The Second Hospital of Dalian Medical University, Dalian, China

**Keywords:** lung squamous cell carcinoma, lactic acid metabolism, intratumor microbiome, immunotherapy, machine learning, tumor microenvironment

## Abstract

**Introduction:**

Cancer development is intricately linked with metabolic dysregulation, including lactic acid metabolism (LM), which plays a pivotal role in tumor progression and immune evasion. However, its specific implications in lung squamous cell carcinoma (LUSC) remain unclear.

**Methods:**

We used numerous datasets encompassing bulk and single-cell transcriptome, genome, intratumor microbiome, and digital pathome to systematically investigate the LM patterns in LUSC. Multiple machine learning algorithms were used to generate the LUSC classification. Histopathology image-based deep learning model was used to predict the classification. Casual mediation analysis was conducted to uncover the association among intratumor microbiota, LM, and immunity.

**Results:**

Two LM-based subtypes were discovered endowed with distinct clinical outcomes and biological peculiarities, such as overall survival, somatic mutations, and intratumor microbiota structure. Moreover, the histopathology image-based deep learning model accurately predicted our LM-based LUSC taxonomy, significantly improving its clinical utility. Machine learning models based on seven LM-related genes (*CHEK2, LIPT1, TUFM, NDUFA10, AGK, PNPLA2,* and *GFM1*) accurately predicted immunotherapy outcomes for multiple cancer types, including LUSC, and outperformed other currently known biomarkers. Furthermore, mediation analysis identified potential association pathways involving tumor-resident microbes, LM-related gene signatures, and antitumor immune cells.

**Discussion:**

Overall, this study advanced the understanding of the relationship between LM patterns and LUSC tumor biology, as well as its potential clinical implications, which might advance the tailored management of LUSC.

## Introduction

Lung cancer is the leading cause of cancer-related deaths, with an estimated 1.0 million fatalities in both the USA and China in 2022 (1). Among non-small cell lung cancers (NSCLC), lung squamous cell carcinoma (LUSC), the second most common subtype, accounts for approximately 30% of all cases (2, 3). Patients with LUSC have limited treatment options beyond chemotherapy, primarily due to the absence of approved genetic alterations that can be targeted with specific therapies (4, 5). Immune checkpoint inhibitor (ICI) therapy, which targets PD-1/PD-L1 and/or CTLA-4, has significantly enhanced the survival rates of patients with LUSC (6, 7). The importance of stratifying patients who are responsive to ICI therapy is highlighted by the bottlenecks encountered in clinical practice, including a low response rate, immune-related adverse events, both primary and acquired resistance, as well as the economic burden associated with this treatment (8).

Lactate secretion is widely recognized as a hallmark metabolic feature of cancer, often termed the Warburg effect (9), which describes cancer cells’ propensity to derive energy through glycolysis even in aerobic conditions, leading to increased lactate production. In the context of LUSC, lactic acid metabolism (LM) and the resulting lactic acidosis within the TME play crucial roles in shaping the tumor ecosystem. Lactate influences intracellular and extracellular signaling pathways within tumor cells (10), enhancing lactate shuttling, bolstering resistance to oxidative stress, and promoting lactylation (11), a post-translational modification that bridges metabolism and epigenetics. Moreover, lactate interacts with various immune cell populations within the TME, modulating processes such as cell differentiation, immune responses, immune surveillance, and therapeutic efficacy (12–15). The lactate shuttle, facilitating the exchange of lactate between hypoxic and aerobic regions of the tumor, is pivotal for tumor monitoring and adaptation to changing metabolic conditions (10). The complex interplay between lactate and immune cells, as well as stromal/endothelial cells, supports basement membrane remodeling, epithelial-mesenchymal transition (EMT), metabolic reprogramming, angiogenesis, and drug resistance, further complicating the therapeutic landscape of LUSC (16, 17). However, in LUSC, the regulatory role of LM on the TME, particularly its immune components, remains poorly understood.

Growing evidence suggests that microbes can reside in tumor cells and immune cells and influence the state of the TME (18, 19). Lactic acid bacteria in the TME can alter tumor metabolism and lactate signaling pathways, leading to therapeutic resistance, which is expected to be a therapeutic target for various cancers (20). Besides, one study discovered two subtypes based on lung-resident microbial score endowed with distinct glycolysis-lactate patterns and clinical outcomes (21). Gu at al., reported that in colorectal cancer liver metastasis, enhanced lactate production by *E. coli* promotes M2 macrophage polarization by inhibiting NF-κB signaling, a process mediated through RIG-I lactylation (22). However, considering the heterogeneity of LUSC and the complex interaction between intratumor microbiome and host, the biological links among tumor-resident microbes, LM, and tumor immunity have not been fully elucidated.

In this study, we hypothesized that tumor-resident microbes regulate the expression pattern of LM-related genes, which further affect tumor immunity. Thus, we aimed to investigate the potential role of LM-related genes in biological peculiarities and clinical outcomes of LUSC and to unravel the crosstalk pattern among intratumor microbes, LM-related genes, and immunity.

## Methods

### Construction of the LM signature

First, 228 LM-related genes were collected from published literature (23). Univariate Cox analysis was performed based on the 228 LM-related genes to identify the overall survival (OS)-related genes in TCGA-LUSC. Then, least absolute shrinkage and selection operator (LASSO) regression was conducted to further screen genes. Subsequently, Friends and random survival forest (RSF) algorithms were employed to identify the top 10 genes of importance, respectively. Last, an intersection was conducted to obtain the final genes, called LM.Sig.

### Data acquisition

In survival analysis, the transcriptome and clinical information of LUSC samples from The Cancer Genome Atlas (TCGA) (https://portal.gdc.cancer.gov/) were used to develop the prognostic model. GSE73403 and GSE37745 from the Gene Expression Omnibus (GEO) database (http://www.ncbi.nlm.nih.gov/geo/) were used to independently validate the performance of the prognostic model. GSE33479 was used to validate the expression of LM.Sig in tumor and normal tissues.

In immune analysis, GSE126044, GSE135222, and GSE166449 were used to validate the association between the expression of LM.Sig and immunotherapy efficacy. These three datasets were combined and processed with batch correction with Combat algorithm with the R package “sva”. To develop and rigorously assess a robust LM.Sig-based classifier for predicting the response to immunotherapy, we comprehensively collected 11 cohort datasets consisting of pre-treatment samples treated with immune checkpoint inhibitors (ICIs). The 11 cohorts included a total of 870 patients (308 responders, 729 non-responders) with 6 cancer types, including glioblastoma (GBM, n = 1), renal cell carcinoma (RCC, n = 1), non-small cell lung cancer (NSCLC, n = 3), skin cutaneous melanoma (SKCM, n = 3), gastric adenocarcinoma (STAD, n = 1), bladder urothelial carcinoma (BLCA, n = 3). Regarding the treatment of ICIs, all cohorts were anti-PD-1 except one melanoma cohort was anti-CTLA4. The Combat algorithm, implemented through the R package “sva”, was used to remove the batch effects. Five of 11 cohorts, named Hugo_SKCM_aPD1 (n=28), Kim_LUSC_aPD1 (n=27), IMvigor210_BLCA_aPD1 (n=298), Zhao_GBM_aPD1 (n=34) and Kim_STAD_aPD1 (n=78), were utilized as the independent testing dataset (n = 465). The others (n = 572) were randomly split into two datasets, used as the training dataset (70%, n = 400) and validation dataset (30%, n = 172). **Supplementary Table 1** summarized the detailed information on these ICI cohorts. Besides, GSE148071, a single-cell RNA-sequencing (scRNA-seq) cohort consisting of 18 patients with lung adenocarcinoma (LUAD), 18 patients with LUSC, and six patients with NSCLC, was used to investigate the expression pattern of LM.Sig at the resolution of single-cell level.

### Molecular subtyping, construction and validation of the prognostic model

Multivariate Cox regression analysis was conducted based on the expression of seven LM.Sig and OS for samples in TCGA-LUSC. The formula for calculating the sample risk score was: risk score = PNPLA2*0.0092-CHEK2*0.0239-LIPT1*0.1272-TUFM*0.0029-NDUFA10*0.0152-AGK*0.0322-GFM1*0.0137. GSE73403 and GSE37745 were utilized to independently validate the performance of model. Samples were divided into two groups (high- and low-risk) based on the median of risk score. Subsequently, survival analysis was performed on these two groups. In addition, the protein expression levels of LM.Sig in lung tumor tissues and normal tissues were validated using immunohistochemistry (IHC) staining images, which were obtained from the human protein atlas (HPA) database (https://www.proteinatlas.org/).

### Immune landscape analysis

ESTIMATE algorithm was conducted to calculate the stromal score, immune score, and tumor purity (24). The Tumor Immune Dysfunction and Exclusion (TIDE) score, dysfunction, exclusion and other immune-related indicators were calculated online (http://tide.dfci.harvard.edu/) (25). 98 immune contexture signatures, 1314 immune-related genes, and gene signatures involving in seven steps of antitumor immune cell were obtained from published literature (26). Besides, TIMER (27), CIBERSORT (28), quanTIseq (29), MCP-counter (30), xCell (31), and EPIC (32) were used to estimate the abundance of various immune cells based on the gene expression matrix. Besdies, the Gene Set Variation Analysis (GSVA) score of LM-related genes was calculated with R package “GSVA”.

### Characterization of somatic mutations and drug sensitivity analysis

To describe the somatic mutations in patients belonging to various subtypes, MAF files of patients were obtained from the TCGA database and subsequently analyzed and visualized utilizing the R package “maftools” (33). The predicted half maximal inhibitory concentrations (IC50) for 198 commonly used antitumor drugs in LUSC were computed using the R package “oncoPredict” (34).

### WSI-based deep-learning model: CLAM

Multiple-instance learning (MIL) represents a paradigm of weakly supervised learning where data is organized into bags of instances. Given the whole slide image (WSI)-level label (along with annotated tumor regions of interest in the experiments), MIL models possess the capability to predict labels for unseen WSIs by considering the most predictive patches. Clustering-constrained-attention multiple-instance learning (CLAM) is a recently introduced, advanced MIL approach tailored specifically for digital pathology, with its code accessible at https://github.com/mahmoodlab/CLAM (35). Its attention mechanism enables the model to automatically concentrate on representative patches.

The initial stage entails extracting features utilizing a ResNet50 model, which has been modified and pre-trained on the ImageNet dataset. The first fully connected (FC) layer reduced the features down to 512 dimensions, and the subsequent FC layer served as a classifier, generating 2-class scores for each patch. A max-pooling function was then employed on the “Cluster High” class to select the top-1 patch. Subsequently, the scores of this patch were normalized to WSI-level probabilities using the softmax function. For the models investigated, training was performed using a five-fold cross-validation strategy. For each fold, the dataset was randomly partitioned into training (80% of cases) sets and validation (20%) sets. Model performance was further evaluated using the area under the ROC curve (AUC).

CLAM generates interpretable heatmaps, which provide users with a clear visualization of how each tissue area within a WSI contributes to the model’s predictions (35). By examining these heatmaps, pathologists can discern which histological and cytological features carry a high predictive weight. Additionally, we used a previous pipeline to extract the texture features of each WSI of TCGA-LUSC dataset, as detailed in the original article (36).

### Construction and validation of the machine-learning model for predicting the ICI response

To evaluate the predictive accuracy of LM.Sig in predicting ICI response, nine machine-learning techniques were utilized: Naive Bayes (NB), AdaBoost Classification Tree (AdaBoost), Random Forest (RF), extreme gradient boosting (xgbTree), recursive partitioning (Rpart), k-Nearest neighbors (KNN), support vector machine (SVM) model utilizing three kernel functions-linear (svmLinear), polynomial (svmPoly), and radial basis function (svmRadial). Nested cross-validation (CV) was employed as the benchmarking strategy for these methods. The trained models’ performance was assessed using a validation dataset, with the model exhibiting the highest AUC being chosen as the optimal LM.Sig model for predicting ICI response. Subsequently, independent test datasets were employed to further evaluate the performance of this optimal model.

Additionally, we compared the predictive capabilities of the LM.Sig model against 10 previously established ICI response models (**Supplementary Table 2**). This comparison encompassed the validation set, the consolidated testing set, the KIM_STAD set, the KIM_LUSC set, the Zhao_GBM set, the IMvigor210 cohort, and the Hugo_SKCM set.

### Single-cell RNA sequencing data processing and analysis

scRNA-seq data analysis was carried out using the R package “Seurat” (v5.1.0) (37). Cells with more than 5,000 genes or fewer than 200 genes, or more than 20% of mitochondrial genes were removed. The remaining cells were utilized for downstream analysis. The FindVariableGenes function was employed to identify 2,000 genes with high variability. For dimensionality reduction and clustering, the RunPCA and RunUMAP functions were utilized. The optimal number of principal components (PCs), determined by the inflection point observed in the ElbowPlot function, was set to 20. Based on the clustering results, cell types were annotated using a combination of the CellMarker database (http://bio-bigdata.hrbmu.edu.cn/CellMarker/) and previously reported gene markers. The FindAllMarkers function was used to investigate the key genes of each cell subpopulation (min.pct=0.25, logfc.threshold=0.25). The uniform manifold approximation and projection (UMAP) analysis was conducted using Seurat’s built-in RunUMAP function. To calculate the metabolic scores for diverse clusters of cell subtypes, the R package “scMetabolism” was employed, utilizing the single-sample gene set enrichment analysis (ssGSEA) method based on the Kyoto Encyclopedia of Genes and Genomes (KEGG) pathway.

### Mediation analysis

Mediation analysis seeks to assess the process by which an exposure influences an outcome, via a mediator, thereby facilitating the exploration of potential mechanisms underlying the effect of exposure on the outcome. In this study, the mediation analysis focused on the LM-related intratumor microbiota, LM.Sig, and immune cell populations. We first checked whether the intratumor microbial features were associated with the LM.Sig using Spearman correlation (*P* < 0.05). Next, mediation analysis was carried out with interactions between mediator and outcome using the mediate function from R package “mediation” to infer the mediation effect of LM.Sig and the intratumor microbiota on tumor immune infiltration.

### Intratumor microbiome analysis

The intratumor microbiome abundance data was obtained at https://github.com/knightlab-analyses/mycobiome provided by Narunsky-Haziza et al. (38). Narunsky-Haziza et al. systematically analyzed tissue and other samples from tens of thousands of patients with 35 types of cancer, revealing the composition and distribution of microbes in different tumor types. The authors included data from four cohorts: the WIS cohort of the Weizmann Institute of Science, TCGA, Hopkins cohort, and UCSD cohort. In order to control the pollution caused by environment and operation process, for the WIS data, the authors selected 104 paraffin samples and 191 negative controls. For the TCGA data, the authors used computational software to filter the data in various ways, such as comparing the results with those of WIS, the HMP project, and more than 100 other literatures. In our study, we used the intratumor microbiome abundance of samples in TCGA-LUSC.

### Statistical analysis

All tasks related to data processing, statistical analysis, and plotting were performed using R software version 4.4.1. The Kaplan-Meier (K-M) method was used to estimate the OS among subtypes, and the log-rank test was applied to compare these estimates. Mantel test and Procrustes test were performed to examine the correlation between antitumor immune cycles-related genes and LM-related genes. Wilcoxon test was conducted to compare the difference of continuous variables between the two groups. Fisher exact test was utilized to perform statistical analysis on categorical variables. False discovery rate (FDR) test was employed to adjust the p-values, and Spearman correlation analysis was used to determine the correlation. All statistical *p*‐values were two‐sided. Statistical significance was set at *P* < 0.05.

## Results

### Construction of molecular subtypes based on the LM-related genes

**Figure 1:**
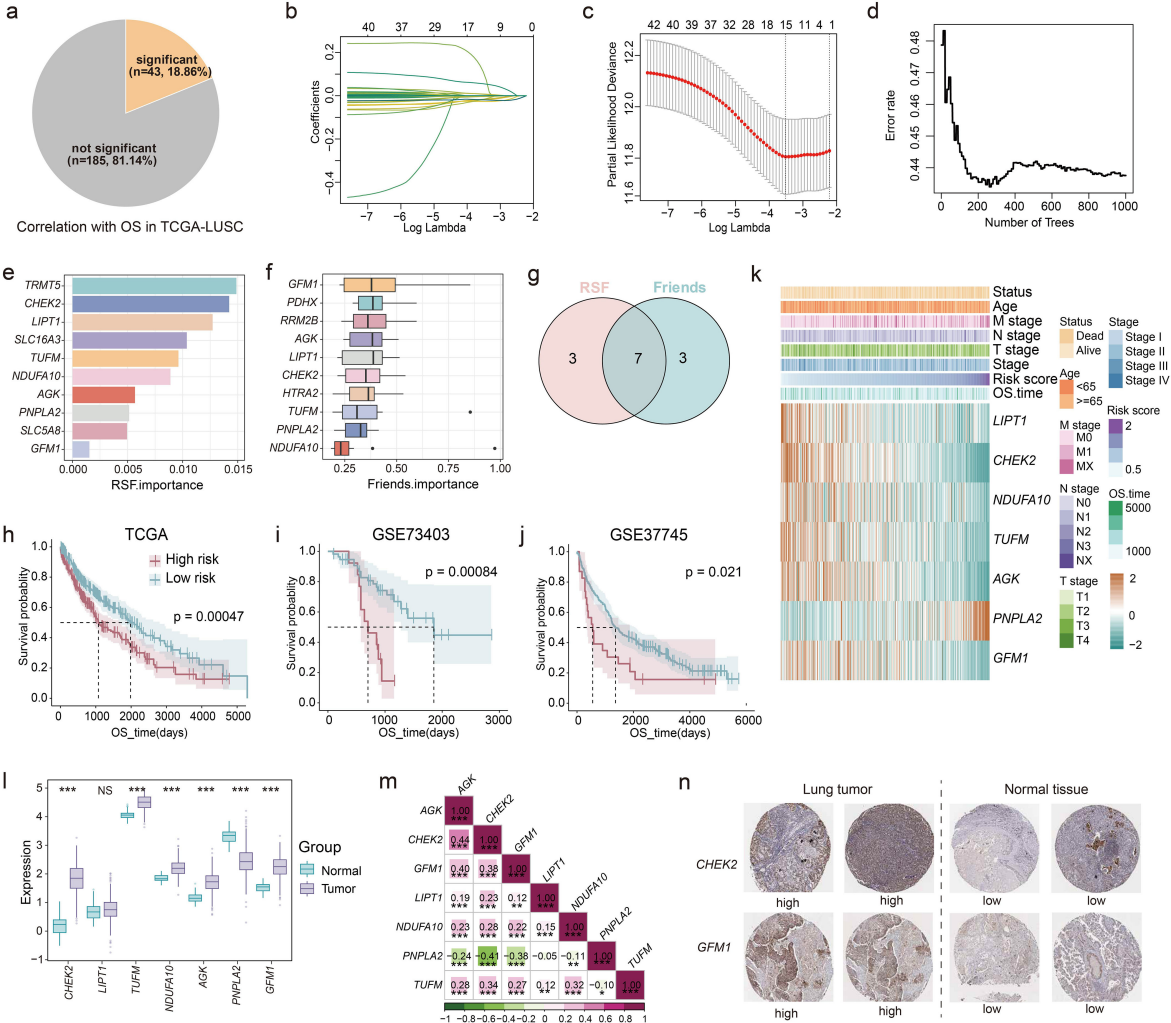
Development of prognostic model based on the LM-related genes. **(a)** The pie chart showing the number and proportion of LM-related genes significantly associated with OS performed by univariate Cox regression in TCGA-LUSC. **(b, c)** Lasso further screening the prognostic genes. **(d, e)** RSF identifying the top 10 most important genes based on the result of lasso. **(f)** Friends analysis identifying the top 10 most important genes based on the result of lasso. **(g)** The intersection of the results of RSF and Friends analysis. K-M curves of OS stratified by risk score in **(h)** TCGA-LUSC, **(i)** GSE73403, and **(j)** GSE37745. Log-rank test was used to generate the *p* values. **(k)** Heatmap showing the association between the expression of genes in LM.Sig and risk score, as well as some clinical indicators in TCGA-LUSC. **(l)** Boxplot showing the differences in the expression of genes in LM.Sig between tumor and normal tissues. Wilcoxon test was conducted to generate the *p* values. **(m)** Spearman correlation between genes in LM.Sig. **(n)** Representative IHC staining images of CHEK2 and GFM1 in lung tumors and normal tissues. *P < 0.05, **P < 0.01, ***P < 0.001. NS, not significant.

First, 228 LM-related genes were obtained from previous literature (23). Functional enrichment showed that these genes were mainly involved in various metabolism- and energy synthesis-related pathways, such as citric acid cycle (TCA cycle), adenosine triphosphate (ATP) biosynthetic process and pyruvate metabolism (**Supplementary Figure 1**). We performed univariate Cox regression analysis based on the expression of LM-related genes and OS of patients in TCGA-LUSC (**Figure 1a**). Results showed that a total of 43 genes (43/228, 18.86%) were significantly associated with OS. Subsequently, lasso regression was used to further screen the genes, and 15 genes were retained (**Figures 1b, c**; **Supplementary Figure 2**). To further identify key genes, RSF was conducted on the 15 LM-related genes, and identified the top 10 characteristic genes, including *TRMT5*, *CHEK2*, *LIPT1*, *SLC16A3*, *TUFM*, *NDUFA10*, *AGK*, *PNPLA2*, *SLC5A8* and *GFM1* (**Figures 1d, e**). Additionally, the Friends algorithm selected 10 critical genes: *GFM1*, *PDHX*, *RRM2B*, *AGK*, *LIPT1*, *CHEK2*, *HTRA2*, *TUFM*, *PNPLA2* and *NDUFA10* (**Figure 1f**). The intersection results from these two algorithms highlighted seven core genes (LM.Sig): *GFM1*, *AGK*, *LIPT1*, *CHEK2*, *TUFM*, *PNPLA2* and *NDUFA10* (**Figure 1g**).

Then, we carried out multivariate Cox regression analysis based on the expression of the LM.Sig and OS in TCGA-LUSC. The risk score of each patient was calculated (the computational formula is provided in the methods) and patients were stratified into two groups based on the median of risk score showing significant difference in OS (**Figure 1h**). Furthermore, GSE73403 and GSE37745 were used to independently validate the performance of our prognostic model (**Figures 1i, j**). We also investigated the association of the LM.Sig with clinical information, and the results showed that gene *PNPLA2* correlated with advanced tumor progression and adverse prognosis, while the other six genes exhibited the opposite trend (**Figure 1k**). The risk score was significantly associated with the vital status and N stage (**Supplementary Figure 3**). Moreover, *PNPLA2* showed an overexpression in normal tissues than that in tumor tissues, whereas all the other genes, except *LIPT1*, were up-regulated in the tumor tissues (**Figure 1l**). An external validation also verified these results (**Supplementary Figure 4**). Correlation analysis showed that the expression of *PNPLA2* was negatively correlated with other genes, while other genes were positively correlated (**Figure 1m**). Besides, we also validated the expression levels of LM.Sig using IHC staining, and the results indicated that the expression levels of CHEK2 and GFM1 were downregulated in normal lung tissues compared to tumor tissues (**Figure 1n**). Validation of other genes were shown in **Supplementary Figure 5**.

Collectively, we successfully develop a robust machine-learning model for predicting LUSC prognosis based on the LM.Sig, which can facilitate the stratified management of patients with LUSC.

### Immune landscape of the different molecular subtypes

**Figure 2:**
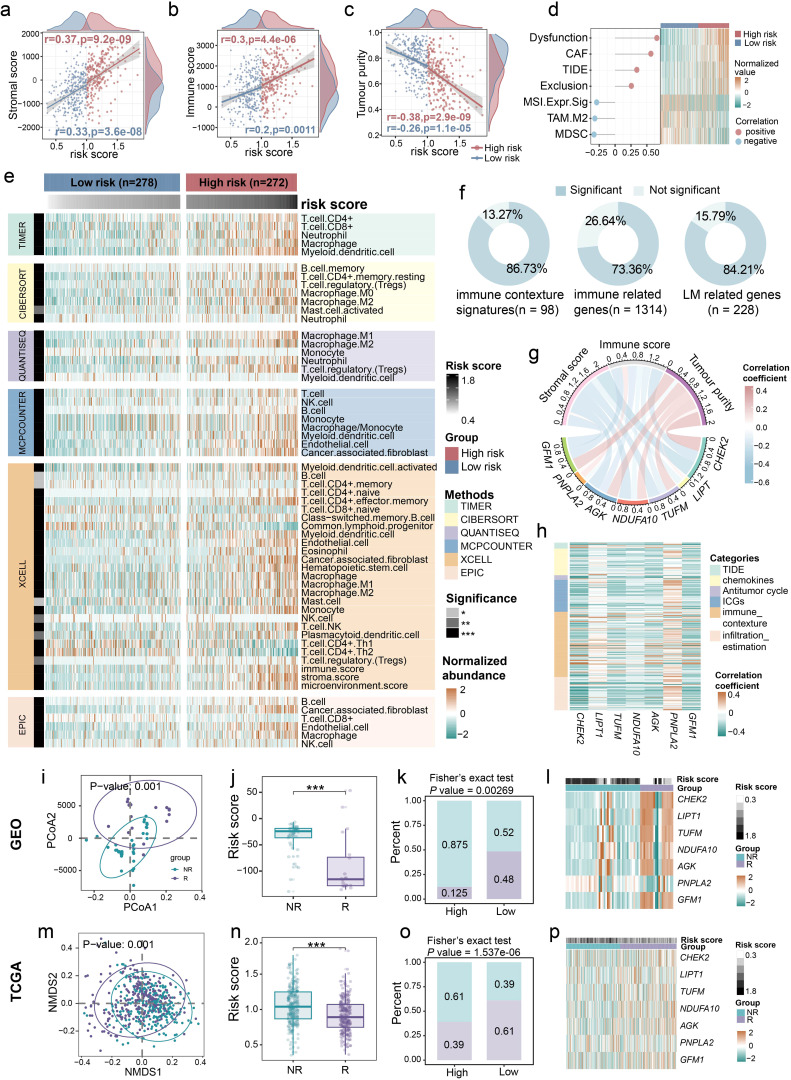
Close relationship between LM.Sig and immune landscape in LUSC. Associations between the risk score and **(a)** stromal score, **(b)** immune score, as well as **(c)** tumor purity. **(d)** The graph on the left showing the correlation between the risk score and immune-related indicators, and the heatmap on the right showing the differences in these indicators between the high- and low-risk group. **(e)** Heatmap showing the differences in the abundance of various immune cells between the high- and low-risk group. The row annotations indicate the method of calculating immune cell abundance and the significance level of the difference in immune cell abundance between the two groups, respectively. The column annotations represent the grouping and risk score of the sample, respectively. **(f)** Pie charts showing the proportions of signatures exhibiting significant difference between the two groups in 98 immune contexture signatures, 1,314 immune-related genes, and 228 LM-related genes, respectively. **(g)** The correlation between the expression of seven genes in LM.Sig and stromal score, immune score, as well as tumor purity. **(h)** Heatmap showing the Spearman correlation between the 7-genes and multiple immune-related measurements, including immune escape-related indicators, chemokines, GSVA score of 7-steps of antitumor immune cycle, immune checkpoint genes (ICGs), immune contexture signatures, and immune infiltration profiles. **(i–l)** The correlations between the ICI response and the LM-related genes, as well as risk score in combined cohorts of GSE126044, GSE135222, and GSE166449. **(m–p)** The correlations between the ICI response (estimated by TIDE) and the LM-related genes, as well as risk score in TCGA-LUSC. * *P* < 0.05, ** *P* < 0.01, *** *P* < 0.001.

Considering the link between LM and the tumor immune microenvironment (TIME) (39, 40), we further investigated the relationship between the LM.Sig and immune landscape in LUSC. Procrustes analysis demonstrated significant association of the expression of LM-related genes with antitumor immune cycle (**Supplementary Figure 6a**; R^2^ = 0.48, *P* = 0.001). Meanwhile, substantial correlations were detected between the risk score and all steps of antitumor immune cycle (**Supplementary Figure 6b**). We also observed that the risk score was significantly positively correlated with the stromal and immune score, and was significantly negatively correlated with the tumor purity (**Figures 2a–c**). Besides, we calculated various immune escape-related scores, including T cell dysfunction score, T cell exclusion score, TIDE, and so on (**Figure 2d**). The risk score was positively correlated with the immune escape capacity of tumors and negatively correlated with the abundance of cell types that limit T cell infiltration in tumors, suggesting that ICI efficacy may be poor in the high-risk group. Using six computational algorithms, including CIBERSORT, EPIC, MCP-counter, TIMER, quanTIseq, and xCell, we comprehensively analyzed immune cell composition (**Figure 2e**). Consistent with previous results, the high-risk group exhibited elevated tumor immune infiltration compared to the low-risk group. However, we also observed significantly higher abundance of M2 macrophages and regulatory T cells (Tregs) in the high-risk group, which could underlie the gloomy prognosis in this group (41, 42). Moreover, when considering 98 immunity contexture signatures, we observed that the majority of the genes were significantly associated with the risk score (**Figure 2f**). A similar trend was observed for the 1,314 immune-related genes and 228 LM-related genes.

We also explored the relationship between the LM.Sig and TIME characteristics. All the genes in LM.Sig were strongly correlated with stromal and immune score, and tumor purity (**Figure 2g**; **Supplementary Table 3**). Interestingly, *PNPLA2* was positively correlated with stromal and immune score, and was negatively correlated with tumor purity. The other six genes showed the opposite correlation trends. Additionally, we also observed the close relationship between the LM.Sig and immune escape-related indicators, chemokines, antitumor immune cycle, immune checkpoint genes (ICGs), immune contexture signatures, and immune infiltration (**Figure 2h**). Consistently, the association of *PNPLA2* with these immune parameters and the association of other genes with these parameters were opposite.

Additionally, we evaluated the relationship between LM.Sig and ICI outcomes. Three ICI cohorts of lung cancer, GSE126044, GSE135222 and GSE166449, were combined, including 20 responders (R) and 45 non-responders (NR). First, we observed significant divergence in the expression of LM-related genes between R and NR (**Figure 2i**; PERMANOVA test, *P* = 0.001). The risk score in the NR subgroup was significantly higher than that in the R subgroup (**Figure 2j**; Wilcoxon test, *P* < 0.001). In the low-risk group, the proportion of R was significantly higher compared to the high-risk group (**Figure 2k**; Fisher’s exact test, *P* = 0.00269). The expression of LM.Sig was significantly different between R and NR (**Figure 2l**). Additionally, these results were further validated using TCGA-LUSC cohort, where TIDE scores greater than 0 were treated as NR and less than 0 as R (**Figures 2m–p**). To further validate the potential of our prognostic indicators in pan-cancer immunotherapy, we used seven independent ICI cohorts covering BLCA, SKCM and other cancer types to assess the association between risk scores and treatment outcomes. The results showed that risk scores in the R subgroup were significantly lower than those in the NR subgroup in all cohorts (**Supplementary Figures 7a–g**).

Consequently, our results offer compelling evidence for a highly negative correlation between LM.Sig and the effectiveness of tumor ICI therapy. Specifically, higher LM.Sig-based scores are associated with an decreased likelihood of ICI efficacy.

### scRNA-seq analysis to assess the LM.Sig

**Figure 3:**
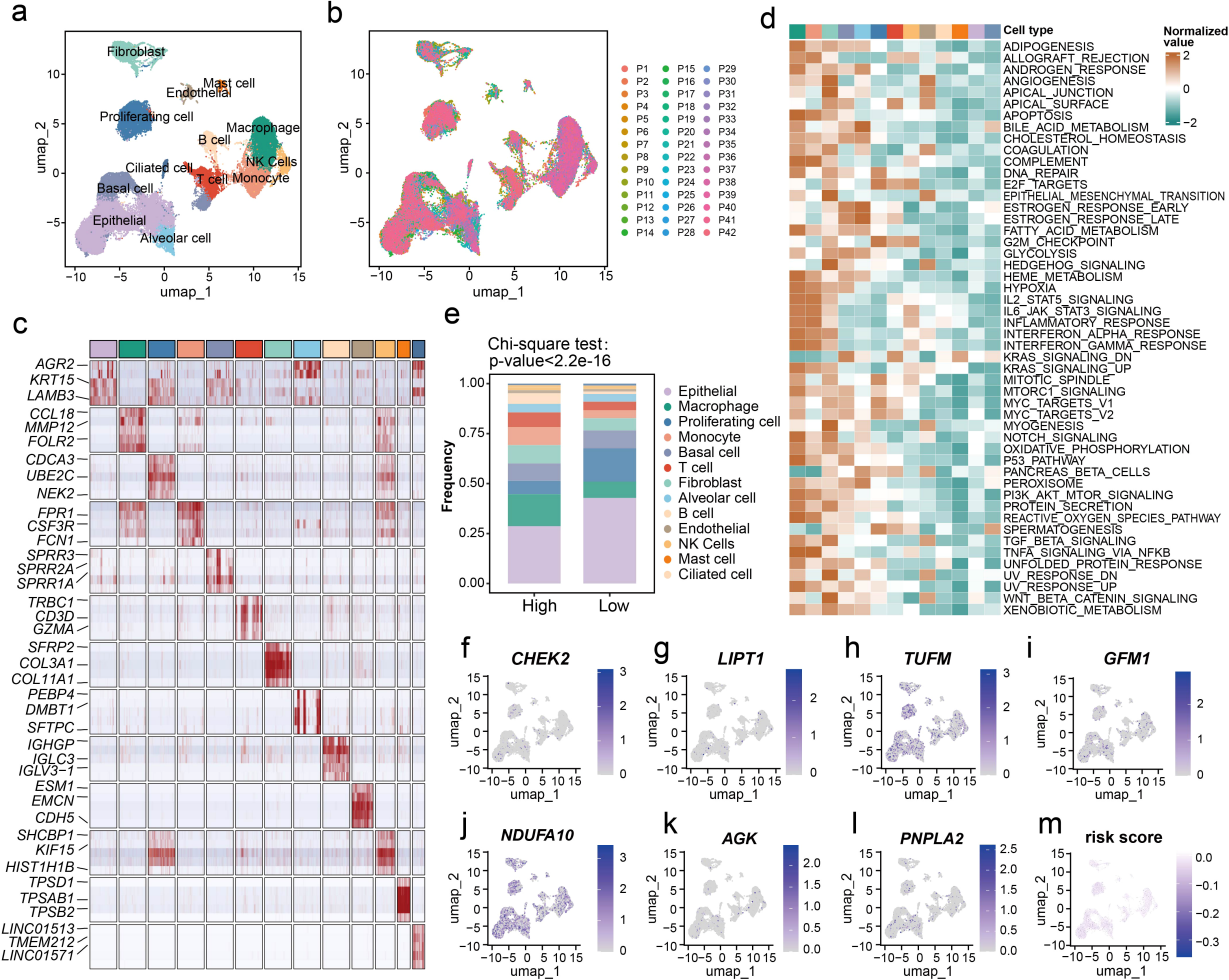
Evaluation of the LM.Sig at the single-cell resolution. **(a)** UMAP plot of all cells, colored by their 13 major cell types. **(b)** UMAP plot of 56,343 cells from 42 patients, colored by patients. **(c)** Heatmap showing the expression of five marker genes in each cell type. The top three significant marker genes of each cell type were labeled on the heatmap. **(d)** Differences in pathway activities scored in each cell type by GSVA. The scores of pathways are normalized. **(e)** The difference in the proportion of the 13 cell types between in the high- and low-risk groups. The chi-square test was used to generate the *P* value. **(f–l)** UMAP plot of the identified cells colored by the expression of LM.Sig. **(m)** UMAP plot of the identified cells colored by the risk score.

We further verified our LM.Sig on the single-cell level using the LUSC scRNA-seq data (GSE148071). The histological and molecular phenotypes, as well as the treatment history were provided in **Supplementary Table 4**. After undergoing multiple quality control and filtering procedures, a total of 56,343 cells were assessed for their transcriptomes. Thirteen major cell types, characterized by their canonical cell markers, were identified and categorized as proliferating cell types, epithelial cells (ciliated cells, basal cells and alveolar cells), immune cell types (T cells, B cells, mast cells, macrophages, natural killer (NK) cells and monocytes) and stromal cell types (fibroblasts and endothelial cells) (**Figure 3a**). We observed that multiple cell types showed significant heterogeneity and patient-specific expression signatures (**Figure 3b**). For each cell type, five marker genes were selected, and except epithelial cells, these marker genes were detected to be widely expressed and prevalent within their respective cell types (**Figure 3c**). We further characterized the functions of different cell types by comparing pathway activities (**Figure 3d**). Consistent with previous study (43), macrophages and monocytes exhibited upregulation of various common pathways, such as pathways involved in apoptosis and inflammatory response, as well as multiple cell proliferation- and immune-related pathways. We also evaluated the activity of multiple metabolism pathways in various cell types, including glycolysis, pyruvate metabolism, glutathione metabolism and oxidative phosphorylation. Elevated ssGSEA enrichment scores of these metabolism pathways were detected in proliferating cells and epithelial cells (**Supplementary Figures 8a–e**).

Furthermore, we applied our prognostic model to this single-cell dataset, dividing patients into two groups. Analysis of single-cell resolution further validated our previous results that the two groups of patients exhibited substantial differences at the level of immune infiltration (**Figure 3e**; Chi-square test, *P* < 2.2e-16). Patients in the high-risk group harbored lower proportion of epithelial cells and higher proportion of macrophages compared with the low-risk group. Additionally, we characterized the expression of the LM.Sig in various cell types (**Figures 3f–l**). Interestingly, we did not observe a clear bias in the expression of these genes in specific cell types, and similarly, a homogeneous risk score across all cell types was also observed (**Figure 3m**).

### Distinct driver genes and activated biological pathways of LM-based subtypes

**Figure 4:**
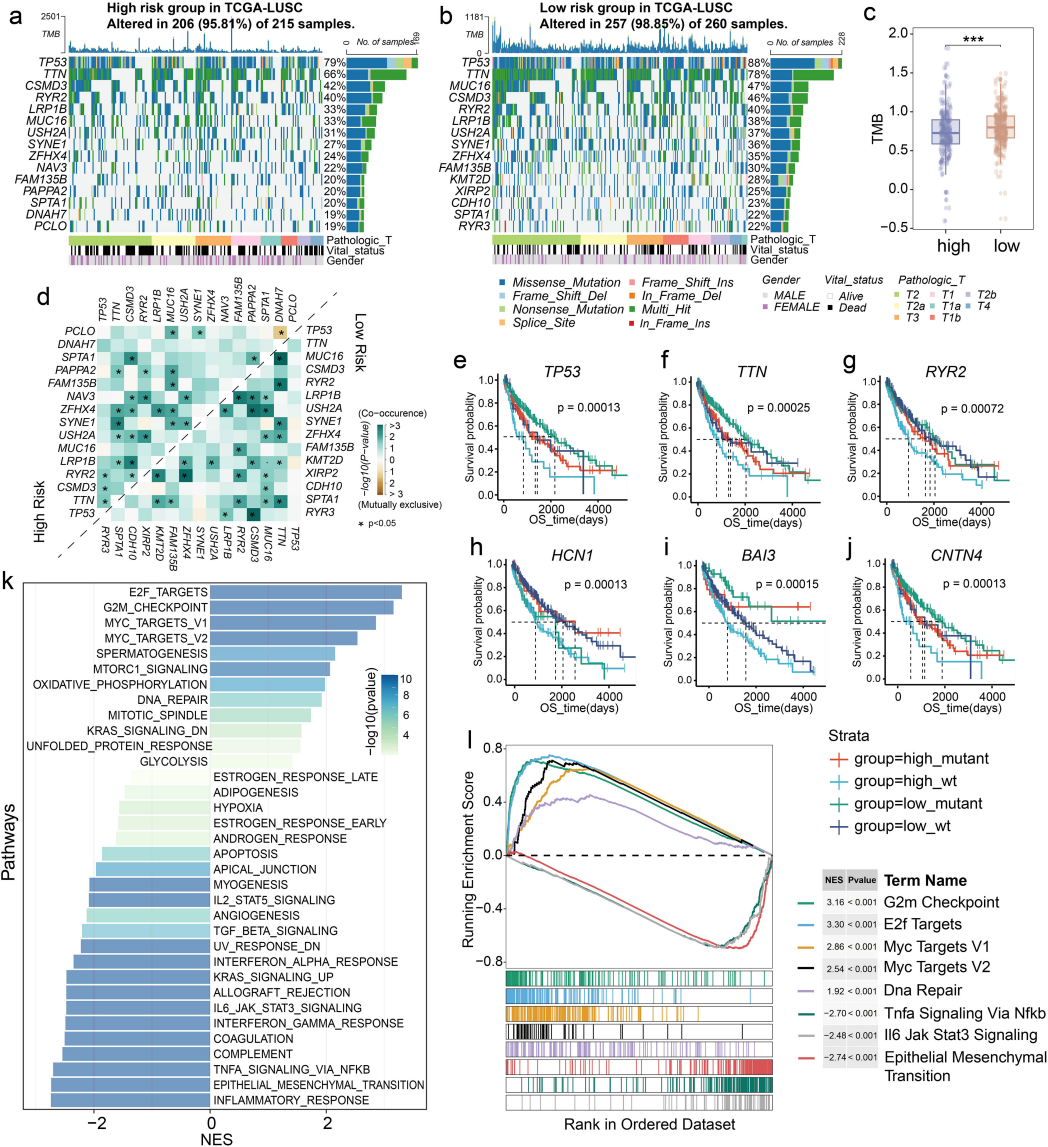
Different driver genes and biological pathways in LM.Sig-based subgroups. Top 15 mutated genes in the **(a)** high- and **(b)** low-risk groups based on TCGA-LUSC cohort. **(c)** Boxplot showing the difference in the TMB value between the two groups. Wilcoxon test was used to generate the *P* value. ***P < 0.001. **(d)** Co-occurrence and mutual exclusivity of top 15 mutated genes in the two groups. *P < 0.05. **(e)** Survival curve showing different risk layers based on risk score and *TP53* mutation status. **(f)** Survival curve showing different risk layers based on risk score and *TTN* mutation status. **(g)** Survival curve showing different risk layers based on risk score and *RYR2* mutation status. **(h)** Survival curve showing different risk layers based on risk score and *HCN1* mutation status. **(i)** Survival curve showing different risk layers based on risk score and *BAI3* mutation status. **(j)** Survival curve showing different risk layers based on risk score and *CNTN4* mutation status. **(k)** Barplot showing the enrichment results of 50 cancer hallmarkers. **(l)** GSEA showing the up- and down-regulated pathways in the two groups.

Next, we sought to investigate the related biological processing of LM.Sig using TCGA-LUSC cohort. Among all mutations present in the LM.Sig, missense mutations were the most prevalent type (**Supplementary Figure 9**). Elevated proportion of patents in the low-risk group had alterations on the driver genes TP53 (88%) and TTN (78%) compared with the high-risk group (**Figures 4a, b**). We detected a significantly higher tumor mutational burden (TMB) in the low-risk group than that in the high-risk group (**Figure 4c**; Wilcoxon test, *P* < 0.001), confirming that patients in the low-risk group were more likely to benefit from immunotherapy. Distinct patterns of gene alteration co-occurrence and mutual exclusivity were observed between the two groups (**Figure 4d**). There were cooperative relationships between the high frequency mutated genes in the two groups. Of these, significant co-alterations of *TP53* and *RYR2*, *TP53* and *CSMD3*, as well as *TP53* and *TTN* were observed only in the high-risk group. *SPTA1* alterations co-occurred at a significant frequency with *MUC16* and *RYR2* only in the low-risk group. Additionally, we observed that *TP53*, *TTN*, *RYR2*, *HCN1*, *BAI3*, and *CNTN4* mutation combined with the LM.Sig-based risk score exhibited distinct risk layers (**Figures 4e–j**).

Then, we analyzed the cancer-related patterns in LM.Sig groups. GSEA analysis indicated that the two groups showed remarkable difference in the activated cancer pathways (**Figure 4k**), such as cell cycle-related pathways including E2F targets, G2M checkpoint and mitotic spindle pathways. Meanwhile, activation of immunomodulatory-related pathways also differed between the two groups, including interferon-gamma response and IL6-JAK-STAT3 signaling. Specifically, compared to the low-risk group, the high-risk group had the suppressed cell cycle pathways and activated EMT, which may explain the poor prognosis of this group (**Figure 4l**; **Supplementary Figure 10**).

### Drug sensitivity of LUSC subtypes

The aforementioned results have deciphered two subtypes with distinct characters, providing directions to subtype‐specific targeted inventions. To better enhance clinical treatment, drug prediction was incorporated for the identification of promising therapeutic agents in two subtypes. Using the “oncoPredict” package, the predicted IC50 values for nearly two hundred FDA-approved anti-cancer drugs were computed. The high- and low-risk groups showed significant difference in the IC50 values of these drugs (**Supplementary Figure 11a**). Moreover, the IC50 values of more than half of the drugs were significantly different between the two groups (**Supplementary Figure 11b**). Then, we identified the top 30 drugs with the most significant differences between the two groups and correlated them with the LM.Sig (**Supplementary Figure 11c**). First, all the expression of genes in LM.Sig were strongly associated with the drug sensitivity. Second, *PNPLA2* was positively correlated with most of these drugs while the other genes were the opposite. Third, the association between the drugs and LM.Sig was consistent with the susceptibility of the drugs in the two groups. Additionally, we identified five drugs that patients with LUSC are most likely to benefit from, including staurosporine, vinblastine, daporinad, dactinomycin, and bortezomib (**Supplementary Figure 11d**). Among the five potential drugs, patients in the high-risk group were more sensitive to staurosporine, while the other four drugs are more appropriate for patients in the low-risk group (**Supplementary Figures 11e–i**).

### Digital pathology predicts LM-based subtypes

**Figure 5:**
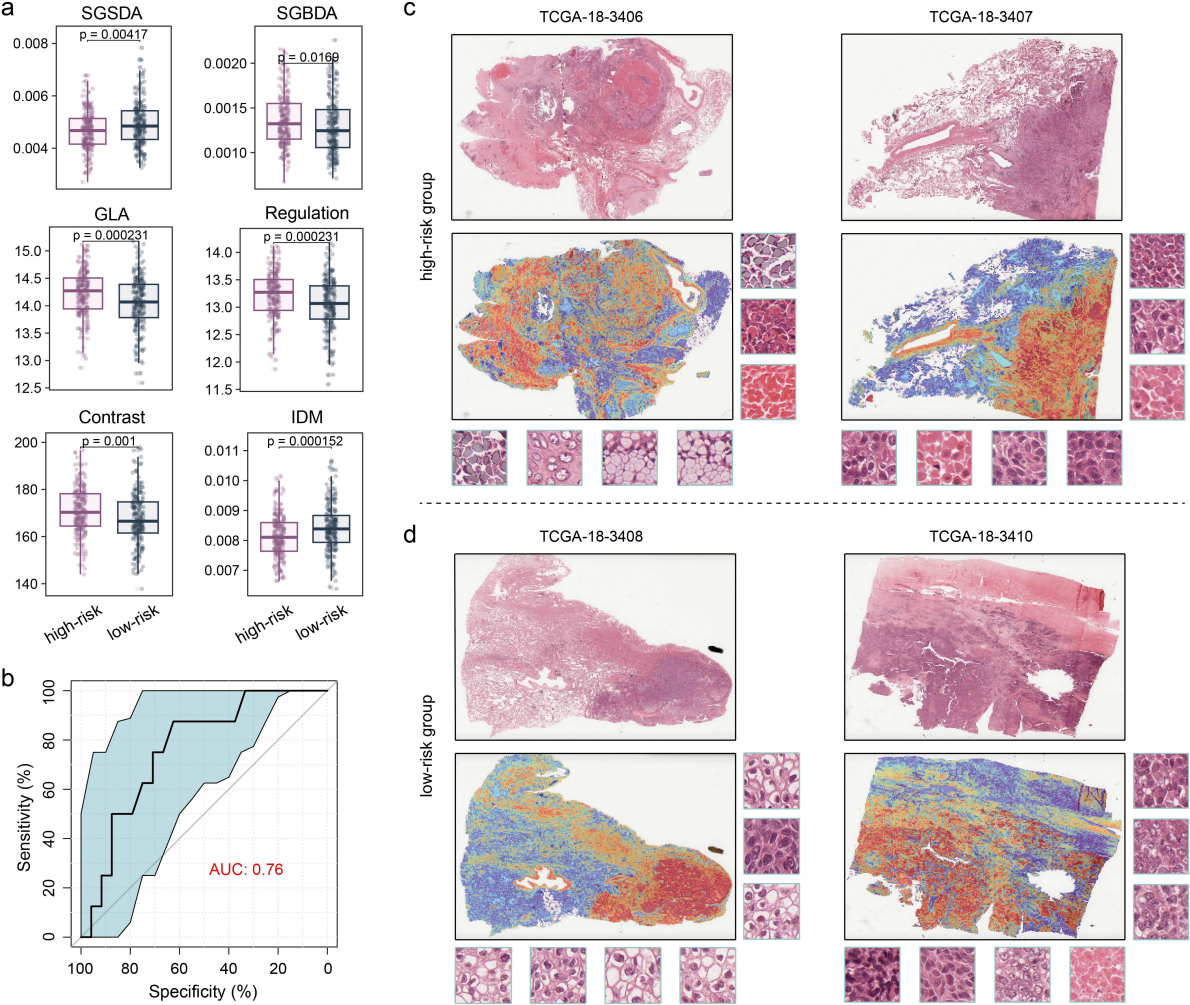
Performance and interpretability of deep-learning model distinguishing the LUSC subtypes. **(a)** Boxplot showing the difference in texture features between the two groups. Wilcoxon test was used to generate the *P* values. **(b)** Performance of the CLAM model in terms of five-fold mean AUC for LUSC subtyping. **(c, d)** Heatmap of whole-slide attention, which corresponds to each slide, was produced by calculating the attention scores for the model’s predicted class across overlapping patches. **(c)** High-risk group. **(d)** Low-risk group.

Because clinical implementation of omics analyses is challenged by high costs, long turnaround times, and complex technical processes, there is a need for cost-effective, fast, and convenient methods to extrapolate the subtypes of this study to improve clinical applicability. First, we extracted the texture features of H&E images of all samples in TCGA-LUSC cohort. We observed significant differences in various texture features between the high- and low-risk groups, including Small Gray-level and Small Detail Advantage (SGSDA), Small Gray-level and Big Detail Advantage (SGBDA), Gray Level Average (GLA), Regulation, Contrast and Inverse Different Moment (IDM) (**Figure 5a**; Wilcoxon test, *P* < 0.05), suggesting profound heterogeneity in digital pathological images of the two LUSC subtypes. Thus, we employed a previous interpretable weakly supervised deep-learning method, called CLAM model (35), to accurately classify whole slides. On the TCGA-LUSC dataset, the model achieved a five-fold mean AUC of o.76 for the LUSC subtyping of high- and low-risk groups (**Figure 5b**). A trained weakly supervised deep-learning classifier offers human-readable interpretability, enabling verification that its predictive foundation aligns with established morphological criteria used by pathologists. This interpretability also aids in analyzing cases where the model fails. Furthermore, whole-slide-level heatmaps enhance clinical diagnoses through artificial intelligence, facilitating human involvement. The CLAM model determines slide-level predictions by pinpointing and amalgamating diagnostically significant regions (with high attention scores) in the WSI, while disregarding less relevant areas (with low attention scores). To visually represent and interpret the importance of each region within the WSI, we created an attention heatmap by converting the model’s attention scores for the predicted class into percentiles and matching these normalized scores to their spatial positions on the original slide. We found that, by utilizing only slide-level labels in weakly supervised learning, the trained CLAM models were generally adept at distinguishing the boundary between tumour and normal tissue (**Figures 5c, d**). Our results demonstrate that the deep-learning model based on digital pathology images performs excellently in distinguishing LUSC subtypes and has good interpretability.

### Development and comparable evaluation of the predictive model for ICI response based on the LM.Sig

**Figure 6:**
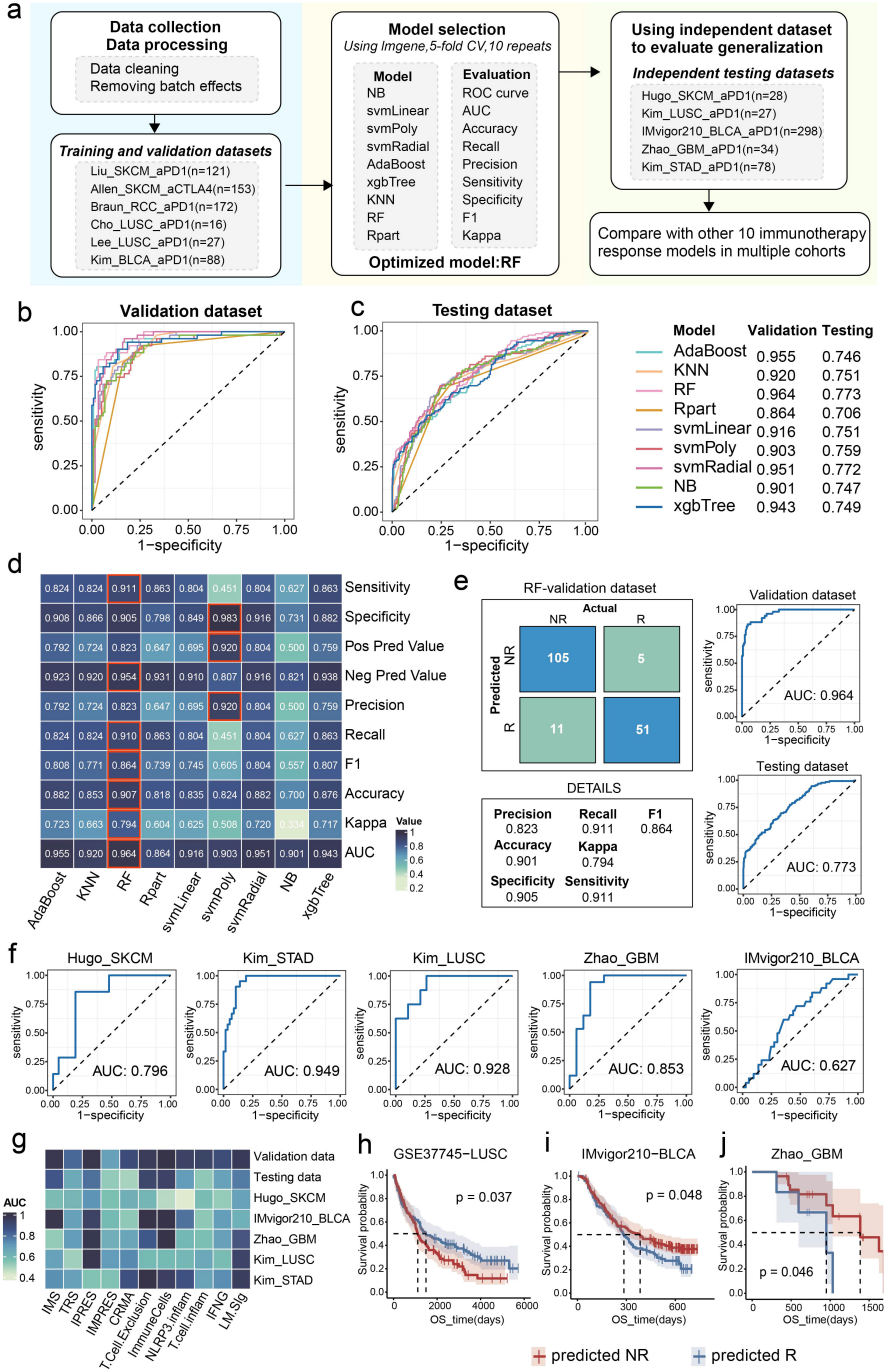
Construction and evaluation of the ICI response predictive model. **(a)** The workflow of development of the predictive model based on the LM.Sig with 9 machine-learning algorithms. The fundamental stages encompass training, validation, and testing of the model. Comparison of the AUC of the multiple models constructed by nine machine-learning algorithms in **(b)** validation and **(c)** testing dataset. **(d)** Heatmap showing the performance of the multiple models developed by nine machine-learning algorithms in validation dataset. **(e)** ROC plots and confusion matrix showing the performance of the optimal LM.Sig model in validation and testing dataset. **(f)** ROC plots showing the performance of the optimal LM.Sig model in individual testing dataset, the AUC in which was range from 0.627 to 0.949. **(g)** Heatmap comparing the performance of the optimal LM.Sig with other 10 immunotherapy response models across multiple cohorts. **(h-j)** K-M curves showing the difference in the OS between the predicted “NR” and predicted “R” by the optimal LM.Sig model.

Given the striking correlation between the LM.Sig and the ICI response, we aimed to investigate the potential predictive utility of the LM.Sig for ICI. We collected 11 bulk-level transcriptomic cohorts treated with ICI. As mentioned previously in the methods section, we employed nine machine-learning algorithms to train models, yielding a total of nine trained models. We subsequently evaluated and compared the AUC of these models in the validation dataset (**Figure 6a**). The AUC ranged from 0.864 (Rpart) to 0.964 (RF) (**Figure 6b**). The model trained with the RF algorithm, with the highest AUC, was selected as the predictive model for ICI response (**Figures 6b, c**). Additionally, in the validation dataset, the model trained with the RF algorithm obtained the highest sensitivity, accuracy, recall, and negative predictive value compared with other models (**Figure 6d**), highlighting its powerful predictive performance. To assess the performance of the optimal model, we tested it in the testing dataset, with AUC value of 0.773 (**Figure 6e**).

Furthermore, we assessed the robustness of the predictive model using independent testing cohorts, with the AUC ranging from 0.627 to 0.949 (**Figure 6f**). Particularly, the predictive model achieved excellent performance in LUSC, with AUC value of 0.928. We conducted a comparison of the performance of the optimally predictive model against ten previously published signatures, revealing that the model based on LM.Sig demonstrated exceptional superiority and maintained consistently high predictive efficacy across a majority of the cohorts (**Figure 6g**). To explore the performance of the predictive model for OS in cohorts treated with ICI, based on the prediction results of the optimal model, patients were divided into two groups representing predicted NR and predicted R, respectively. We subsequently performed a log-rank test and significant differences in the OS were detected in the GSE37745_LUSC (*P* = 0.037), IMvigor_BLCA (*P* = 0.048), and GBM dataset (*P* = 0.046) (**Figures 6h–j**).

In summary, the predictive model utilizing LM.Sig for ICI response exhibited significant robustness and superiority when compared to previously reported signatures across diverse cancer types.

### Crosstalk among the intratumor microbiome, LM, and tumor immunity

**Figure 7:**
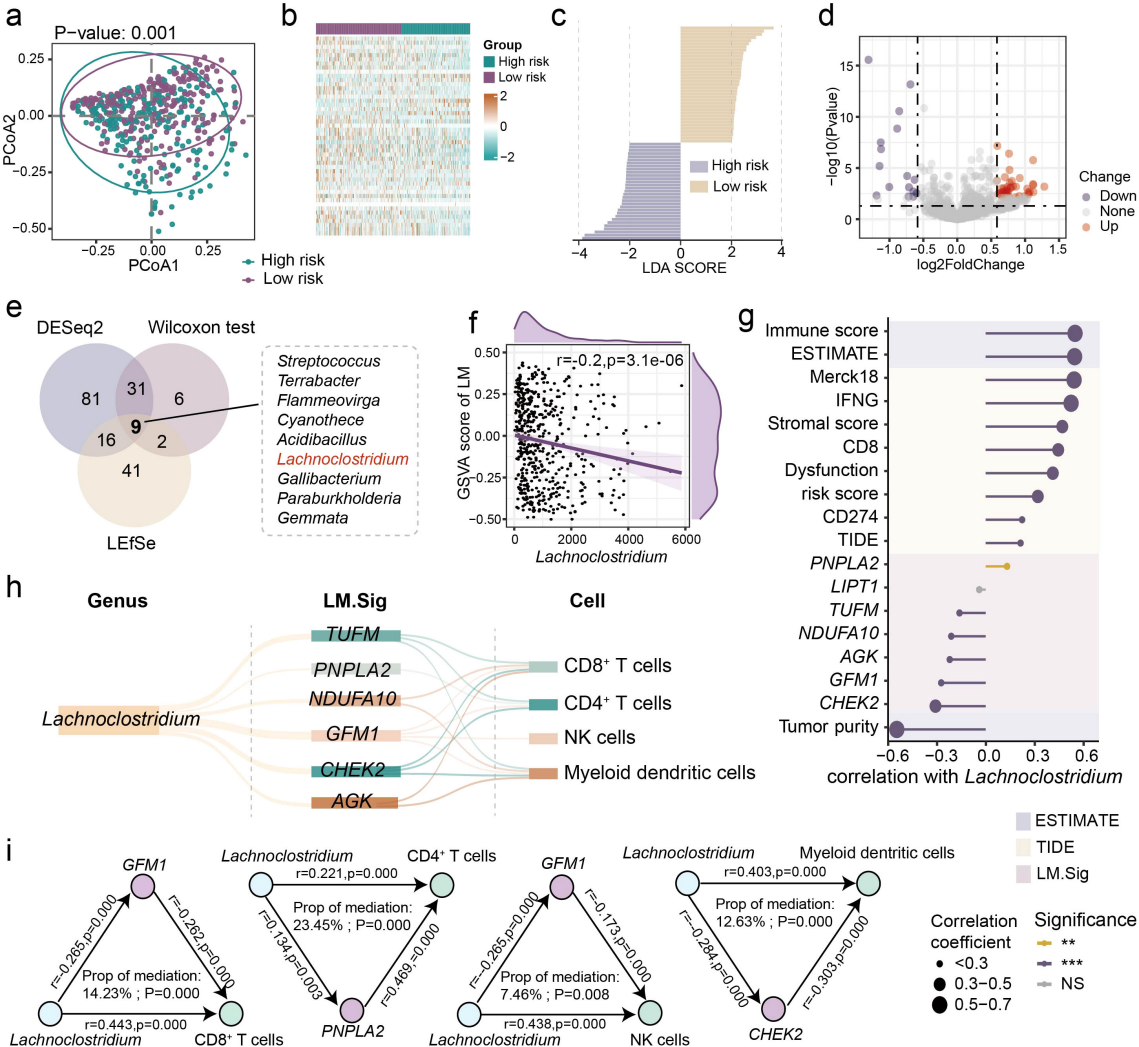
Linkages between the intratumor microbiota, LM.Sig and immunity. **(a)** Principal coordinate analysis (PCoA) showing the divergence in the intratumor microbiota structure between the high- and low-risk groups. PERMANOVA was used to generate the *P* value. **(b)** Heatmap showing the differential microbes between the two groups identified by the Wilcoxon test (adjust *P* value < 0.05). **(c)** LEfSe analysis showing the microbial biomarkers in the two groups with an LDA score > 2. **(d)** Volcano plot showing the differential microbes between the two groups by DeSeq2 with a *P* value < 0.05 and log2(fold change) > 0.5. **(e)** Venn diagram showing the nine key genera identified at the intersection of all three approaches. **(f)** Association between the abundance of genus *Lachnoclostridium* and the GSVA score of LM. **(g)** Correlation between the abundance of *Lachnoclostridium* and immune-related indicators, as well as LM.Sig. The color and size of the circles indicate the statistical significance of the correlation and the correlation coefficient, respectively. **(h)** Parallel coordinates chart showing the 15 mediation effects of LM.Sig that were significant at *P* < 0.05. Shown are genus *Lachnoclostridium* (left), LM.Sig (middle) and immune cells (right). **(i)** Analysis of the effect of the genus *Lachnoclostridium* on immunity as meditated by the LM.Sig. ** *P* < 0.01, *** *P* < 0.001.

Previous study has shown that tumor-resident bacteria can alter tumor metabolism and lactate signaling pathways and cause drug resistance (20). Next, we sought to explore the linkages between intratumor microbiota, LM, and immunity in LUSC. We obtained intratumor microbiome abundance data of LUSC from TCGA samples that were subjected to stringent decontamination and quality control provided by other literature (38). The high- and low-risk groups showed significant difference in the microbiota structure (**Figure 7a**; PERMANOVA test, *P* = 0.001). Subsequently, we carried out three approaches to identify the differential microbes between the two groups, including the Wilcoxon test, linear discriminant analysis effect size (LEfSe), and DeSeq2 (**Figures 7b–d**). The cross-referencing results from these three approaches highlighted nine core genera, including *Streptococcus*, *Terrabacter*, *Flammeovirga*, *Cyanothece*, *Acidibacillus*, *Lachnoclostridium*, *Gallibacterium*, *Paraburkholderia* and *Gemmata* (**Figure 7e**). Particularly, of these genera, we observed that the abundance of genus *Lachnoclostridium* was significantly correlated with the GSVA score of LM (**Figure 7f**; *P* = 3.1e-06). Moreover, significant associations were detected between genus *Lachnoclostridium* and immunotherapy-related indicators, LM.Sig, as well as tumor immune characteristics (**Figure 7g**).

Consequently, we applied a bidirectional mediation analysis to evaluate whether the effect of the genus *Lachnoclostridium* on tumor immunity is mediated via LM.Sig. This approach established 15 mediation linkages for the impact of the genus *Lachnoclostridium* on immune cells through the LM.Sig (**Figure 7h**; **Supplementary Table 5**). We observed that the effect of genus *Lachnoclostridium* on CD8^+^ T cells was mediated via *TUFM*, *GFM1*, *CHEK2*, *NDUFA10* and *AGK* (**Figures 7h, i**). We also observed that the effect of genus *Lachnoclostridium* on CD4^+^ T cells was mediated via *TUFM*, *PNPLA2*, *GFM1* and *CHEK2* (**Figures 7h, i**). In addition, the effect of genus *Lachnoclostridium* on NK cells was mediated via *GFM1* (**Figures 7h, i**). Overall, these results demonstrated that specific intratumor microbes can influence the LM of host and further shape the immune landscapes in LUSC tumors.

## Discussion

LUSC comprises approximately 30% of lung cancers and typically exhibits poor responsiveness to various adjuvant therapies, including molecularly targeted treatments (44). Nonetheless, ICI therapies have yielded promising outcomes in treating LUSC, leading to the approval of several drugs by the FDA for immunotherapeutic applications in LUSC (45, 46). The complexity of the composition in the TME and the heterogeneity of the interactions of its internal factors contribute to drug resistance in LUSC patients (47, 48), where lactic acid metabolic reprogramming and tumor-resident microbes are also two non-negligible constituents (49, 50). The lactate generated through aerobic glycolysis in tumors exerts a widespread influence on both the energy metabolism of the tumors themselves and the composition as well as the functionality of immune cells within the TME (51), while intratumor microbes can alter tumor metabolism and lactic acid signaling pathways through metabolites, causing therapeutic resistance of cancer (20). Therefore, there is a fundamental requirement for cross-talk analysis of LM, intratumor microbes, and immune environment to discern the heterogeneity of LUSC, evaluate patient prognosis, and predict the efficacy of ICIs.

Our study focused on the LM-related modifications of seven key genes (LM.Sig)—*GFM1*, *AGK*, *LIPT1*, *CHEK2*, *TUFM*, *PNPLA2* and *NDUFA10*—each of which plays a crucial role in LUSC progression. The expression of *GFM1* was reported to be significantly elevated in LUSC tumor compared with normal tissues (52). *AGK* is involved in the regulation of various signaling pathways and transcription factors, and its increased expression in tumor cells is associated with poor prognosis in multiple cancers (53, 54). *LIPT1*, a cuproptosis-related gene, is a prognostic indicator in NSCLC (55). *CHEK2*, a classic cancer susceptibility gene, whose harmful mutations are associated with multiple types of cancer (56). Downregulation of *TUFM* promotes epithelial-mesenchymal transition (EMT) and invasion in lung cancer cells through a mechanism that involves AMPK-GSK3β signaling (57). Low expression of *PNPLA2*, the gene encoding adipose triglyceride lipase (ATGL), was associated with significantly reduced survival in patients with NSCLC (58). *NDUFA10*, a core gene in prognostic models for multiple cancers, can predict OS of patients (59, 60).

Our LM.Sig-based prognostic model achieved excellent performance in differentiating patient’s OS, and was validated in multiple independent LUSC cohorts. Furthermore, we found two subtypes of LUSC with different levels of prognosis and immune infiltration. Interestingly, we observed that patients in the high-risk group exhibited more abundant immune cell populations in tumor, which could be explained by higher abundance of M2 macrophages and Tregs in this subtype. Extensive studies have shown that M2 macrophages and Tregs are associated with poor prognosis (42, 61, 62). Using TCGA-LUSC and multiple independent cohorts, we demonstrated that patients in the high-risk group had a lower response rate to ICI therapy. Moreover, the expression of LM.Sig was strongly correlated with the outcome of ICI therapy in multiple cohorts. Further research on these seven genes will advance the development of ICI therapy. In summary, our study proposed a robust LM.Sig-based LUSC classification in which the high-risk group presented characteristics of high tumor immune invasion, gloomy prognosis, and poor immunotherapy response.

Additionally, our study discovered two heterogeneous subtypes endowed with distinct intratumor microbiota structure. Using multiple microbial abundance differential analysis methods, we identified the genus *Lachnoclostridium* and found prominent associations with LM and tumor immunity. Consistent with previous studies, Zhang et al. reported that tumors with distinct relative abundances of *Lachnoclostridium* exhibited variations in their response to immunotherapy and sensitivity to potential drug candidates (63). Another study elucidated that tumor-resident *Lachnoclostridium* could indirectly influence bladder tumor immune infiltration by influencing chemokine expression (64). However, the association between LM and bacteria other than lactic acid bacteria (LAB), such as *Bifidobacterium* and *Lactobacillus*, is still unclear, especially in the development of tumors. We found that intratumor *Lachnoclostridium* could modify the LUSC anti-tumor immune landscape by affecting the expression of LM-related genes. Our study can serve as a precursor for hypothesis-driven research to better understand the causational relationship between intratumor microbiota, LM and tumor immunity in LUSC.

Prognostic models for LUSC based on omics data have recently proliferated. One study identified a signature based on T-cell marker genes to predict prognosis of LUSC (65). Zhu et al. integrated bulk RNA-seq, scRNA-seq and clinical features to predict the OS of LUSC (66). Yang et al. developed a risk model based on m6A-related genes to assess prognosis (67). All these models can effectively stratify the prognosis of patients with multiple biological attributes. However, the high cost and complex processing flow of omics data analysis limit the application of current prognostic models in the clinical management of LUSC. Notably, our LUSC classification can be accurately discriminated by a deep-learning model based on pathological images. Compared with omics sequencing, histopathology images of patients are readily available, cost-effective (no pathologists to label), and have a large sample size for training. As the sample of histopathology images of LUSC patients continues to expand, the performance of our deep learning model will continue to improve, and it will have promising applications in clinical-assisted diagnosis in the future.

Considering the striking association between the LM.Sig and tumor immunity, we constructed predictive model for ICI response based on the LM.Sig. First, we selected the optimal prediction model through the training set and validation set, and then verified the optimal model on the independent dataset. More than 10 immunotherapy cohorts comprehensively confirmed the robustness and generalization ability of our model. Interestingly, in addition to LUSC, our predictive model also performed well on other cancer types, such as STAD and GBM. Chen et al. integrated single-cell sequencing and spatial transcriptome sequencing data at the pan-cancer level and revealed associations between LM and immunotherapy for multiple tumors (23). Furthermore, we compared the performance of our predictive model to ten previously published signatures on multiple datasets, and our model was at the leading level.

We acknowledge several limitations in our study. First, all samples included in this study were collected retrospectively, and it is necessary to conduct further validation of our LUSC classification using prospective data. Second, a thorough investigation into the biological mechanisms that underlie the association between LM and tumor ICI therapy is imperative. This should involve experimental validation and functional analysis of pivotal genes implicated in immune evasion and treatment resistance, in order to gain deeper insights into the underlying processes. Third, our intratumor microbiome data were derived from reanalysis of TCGA data, and in the future, patient-paired 16s rRNA gene sequencing, transcriptome sequencing and other omics will be required to systematically reveal biological associations in LUSC.

## Conclusion

In summary, we identified two LUSC subtypes with different biological peculiarities and clinical outcomes based on seven LM-related genes (*CHEK2*, *LIPT1*, *TUFM*, *NDUFA10*, *AGK*, *PNPLA2*, and *GFM1*). Deep learning models based on histopathology images can accurately distinguish between the two subtypes, greatly improving clinical utility. In addition, machine learning models based on these seven genes performed excellently in predicting the efficacy of ICI therapy. Multi-omics analysis show that tumor-resident *Lachnoclostridium* can modify the tumor immune landscape by influencing the expression of LM-related genes. These findings improve our understanding of LUSC heterogeneity and facilitate clinical tailored management and precise treatment of LUSC patients.

## Data Availability

The original contributions presented in the study are included in the article/**Supplementary Material**. Further inquiries can be directed to the corresponding author.
