# Supplementary material for: Multi-omics analysis untangles the crosstalk between intratumor microbiome, lactic acid metabolism and immune status in lung squamous cell carcinoma
Source: Front Immunol. 2025 Jun 11;16:1603822. doi: 10.3389/fimmu.2025.1603822 (PMC12187763; doi:10.3389/fimmu.2025.1603822)
Supplement: Supplementary file 1 [file DataSheet1.docx]

**Multi-omics analysis untangles the crosstalk between intratumor microbiome, lactic acid metabolism and immune status in lung squamous cell carcinoma**

Xun Qiu^1^, Dan Li^1*^

^1^Department of Medical Oncology, The Second Hospital of Dalian Medical University, Dalian, 116021, China

*Correspondence: Dan Li, E-mail: cmulidan@126.com

**Contents**

**Supplementary Figure 1**

**Supplementary Figure 2**

**Supplementary Figure 3**

**Supplementary Figure 4**

**Supplementary Figure 5**

**Supplementary Figure 6**

**Supplementary Figure 7**

**Supplementary Figure 8**

**Supplementary Figure 9**

**Supplementary Figure 10**

**Supplementary Figure 11**

**Supplementary Table 1**

**Supplementary Table 2**

**Supplementary Table 3**

**Supplementary Table 4**

**Supplementary Table 5**


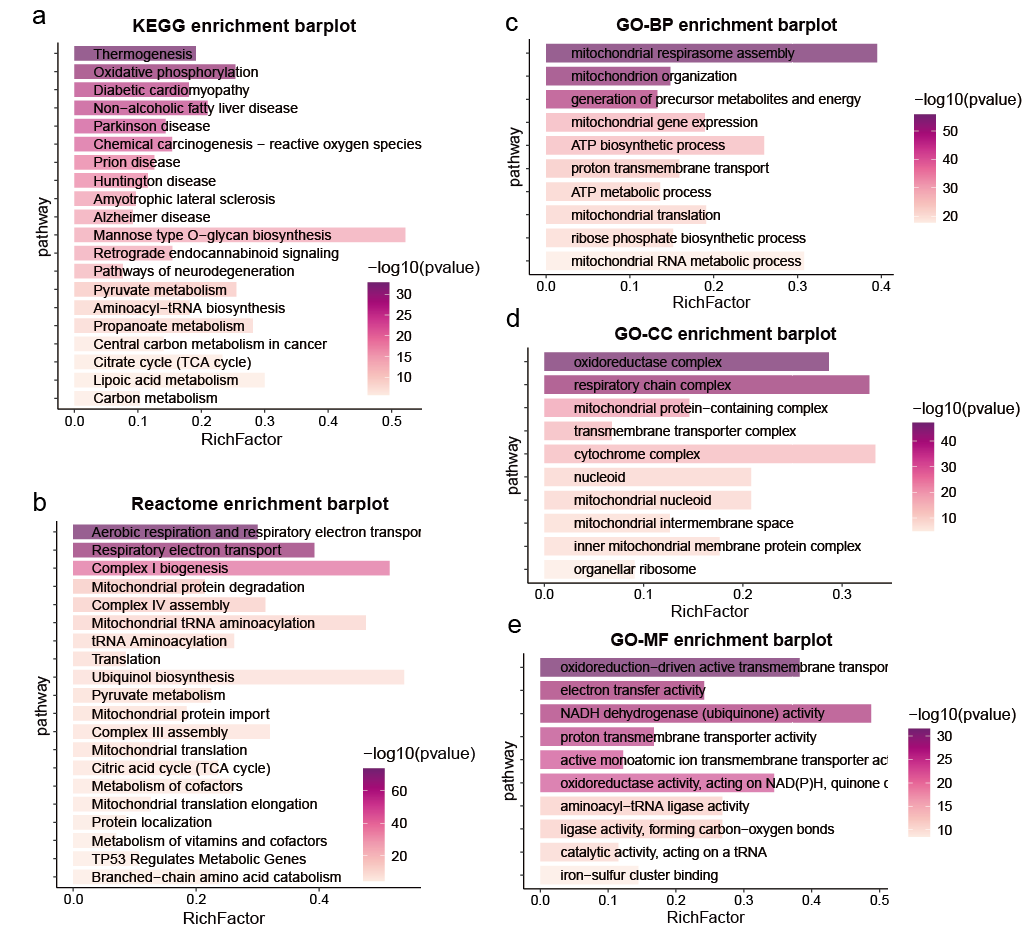


**Supplementary Figure 1. Functional enrichment results of the LM-related genes.** (a) Enrichment results of KEGG. (b) Enrichment results of the Reactome. (c-e) Enrichment results of GO.


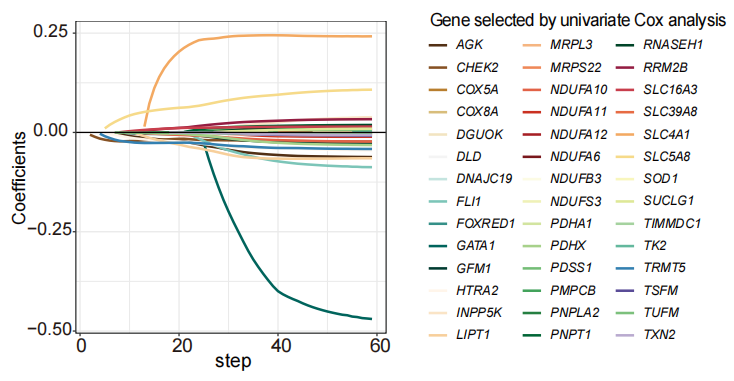


**Supplementary Figure 2. Genes were further screened by the lasso regression analysis.** A total of 43 genes correlated with OS identified by univariate Cox analysis was subjected to the lasso regression.


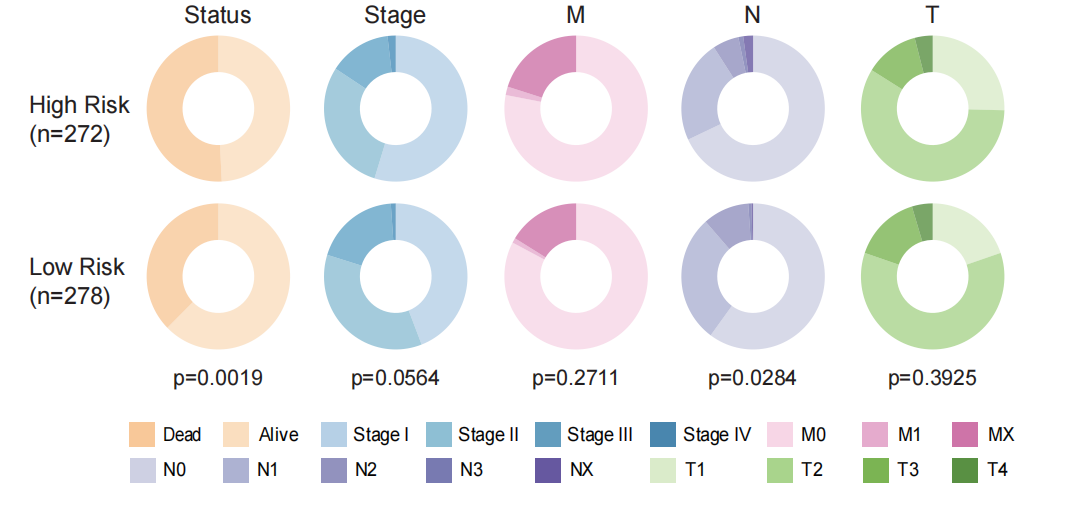


**Supplementary Figure 3. Differences in the clinical indicators between the high- and low-risk.** Fisher exact test was used to performed the statistical test.


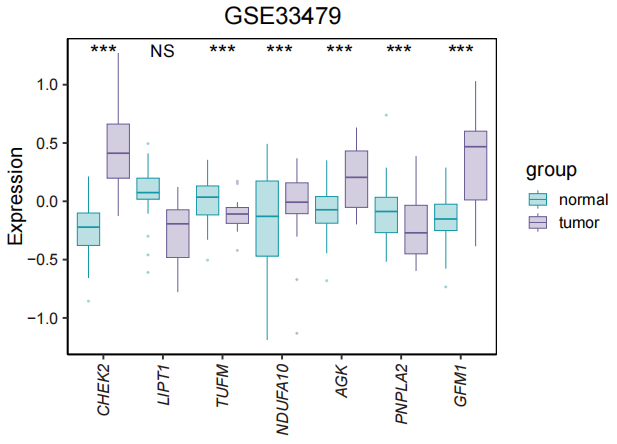


**Supplementary Figure 4. Boxplots showing the differences in the expression of genes in LM.Sig between tumor and normal tissues in GSE33479.** Wilcoxon test was used to generate to the *P* values. NS, not significant. *** *P* < 0.001.


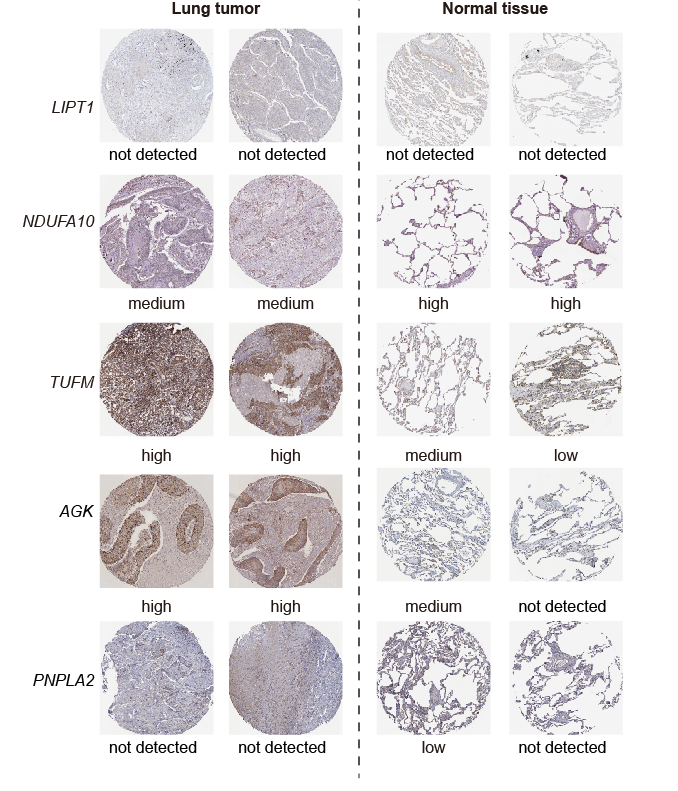


**Supplementary Figure 5. Representative IHC staining images of *AGK*, *LIPT1*, *TUFM*, *PNPLA2* and *NDUFA10* in lung tumors and normal tissues**. Dotted lines indicate lung tumor tissues on the left and normal tissues on the right.


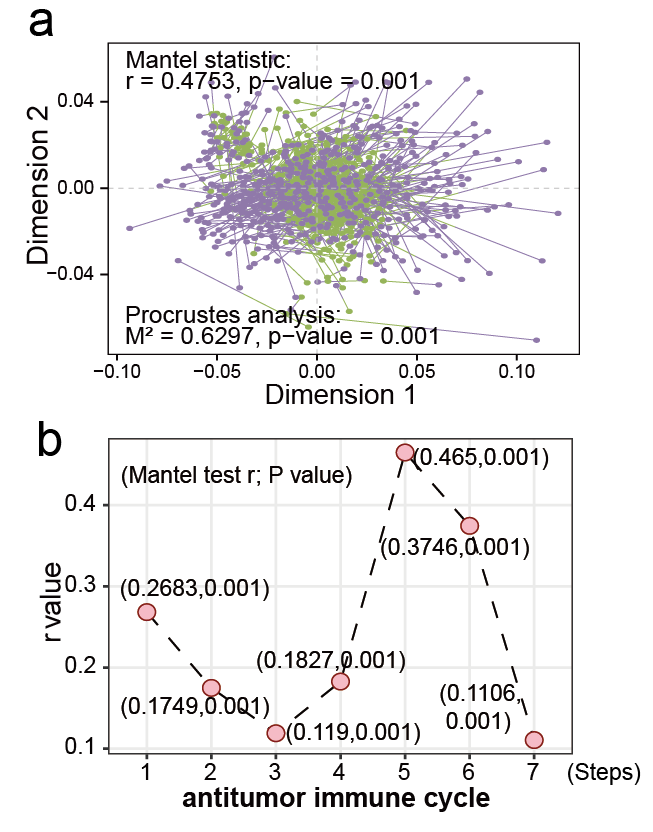


**Supplementary Figure 6. Correlation between the LM and antitumor immune cycle.** (a) Procrustes analysis showing the correlation between the expression of genes in 7-steps of antitumor immune cycle and the expression of LM-related genes. (b) Mantel test showing the correlations between genes in each step and the risk score. Step1: release of cancer cell antigens; step2: cancer antigen presentation; step3: priming and activation; step4: trafficking of immune cells to tumors; step5: infiltration of immune cells into tumors; step6: recognition of cancer cells by T cells; step7: killing of cancer cells.


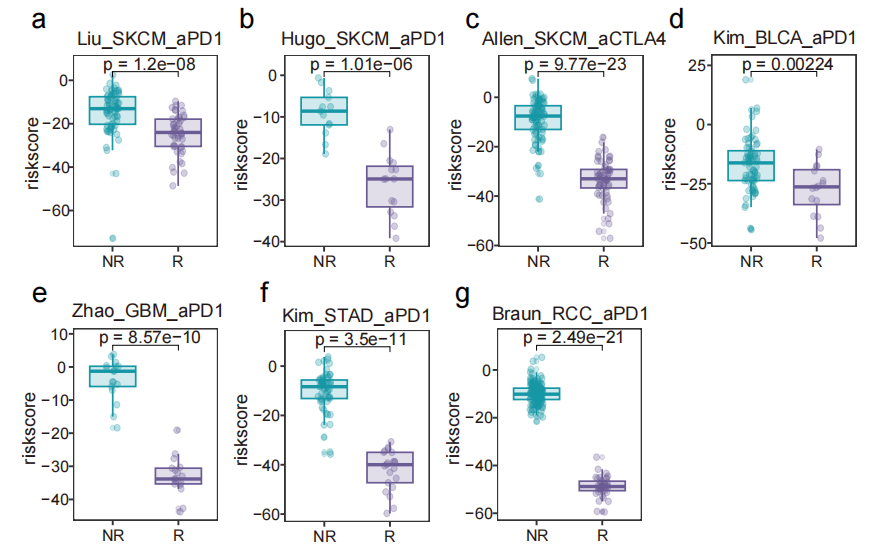


**Supplementary Figure 7. Comparisons of the risk score between the R and NR subgroup in multiple ICI cohorts.** (a-c) SKCM; (d) BLCA; (e) GBM; (f) STAD; (g) RCC. Wilcoxon test was used to generate to the *P* values.


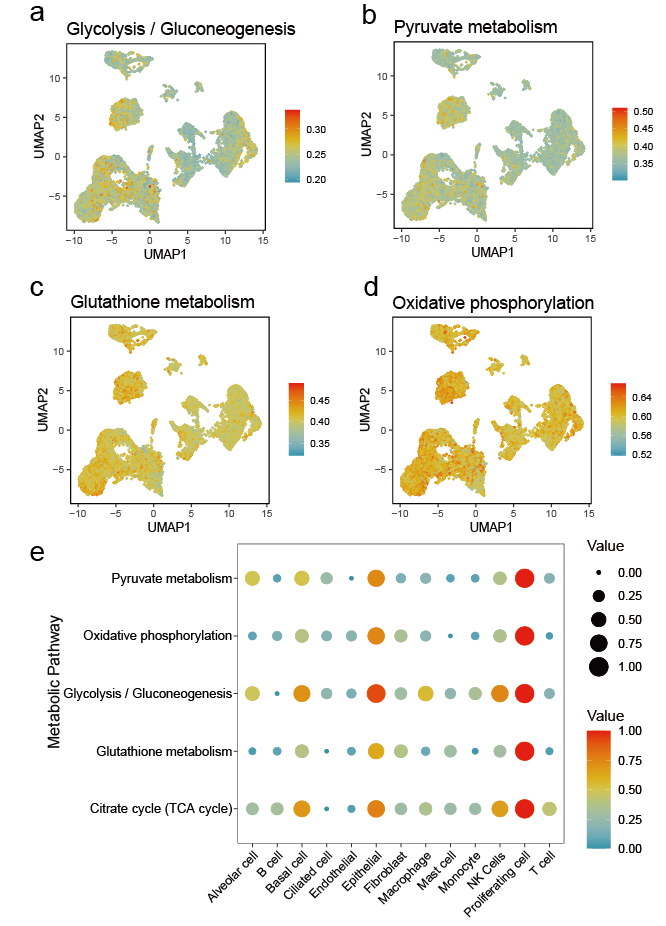


**Supplementary Figure 8. Evaluation of activity of various metabolism pathways at the single cell resolution.** UMAP plots of the identified cells colored by the ssGSEA score of (a) glycolysis, (b) pyruvate metabolism, (c) glutathione metabolism, (d) oxidative phosphorylation. (e) Bubble diagram showing the ssGSEA score of five metabolic pathways in various cell types.


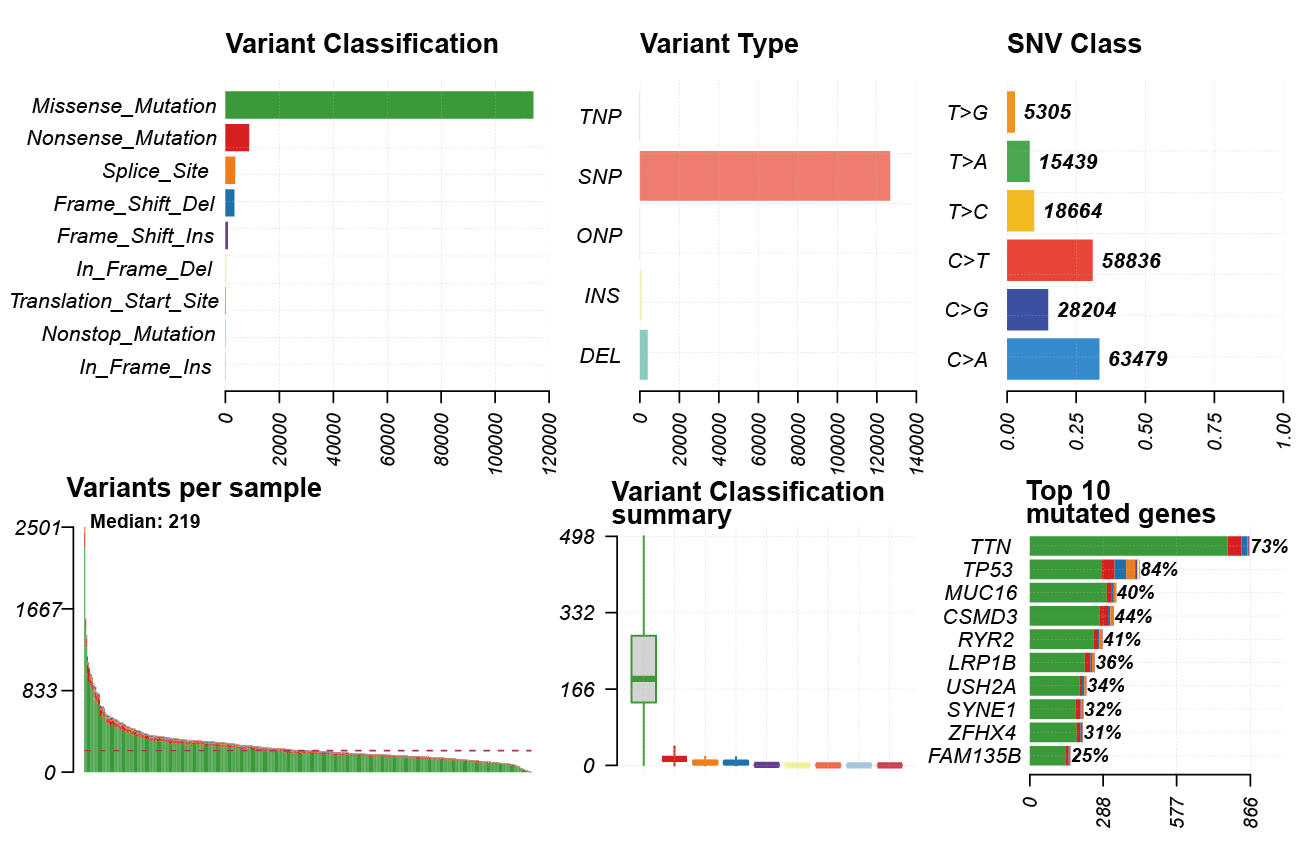


**Supplementary Figure 9. A panel plot presenting a summary of the frequencies of various genomic mutation types.**


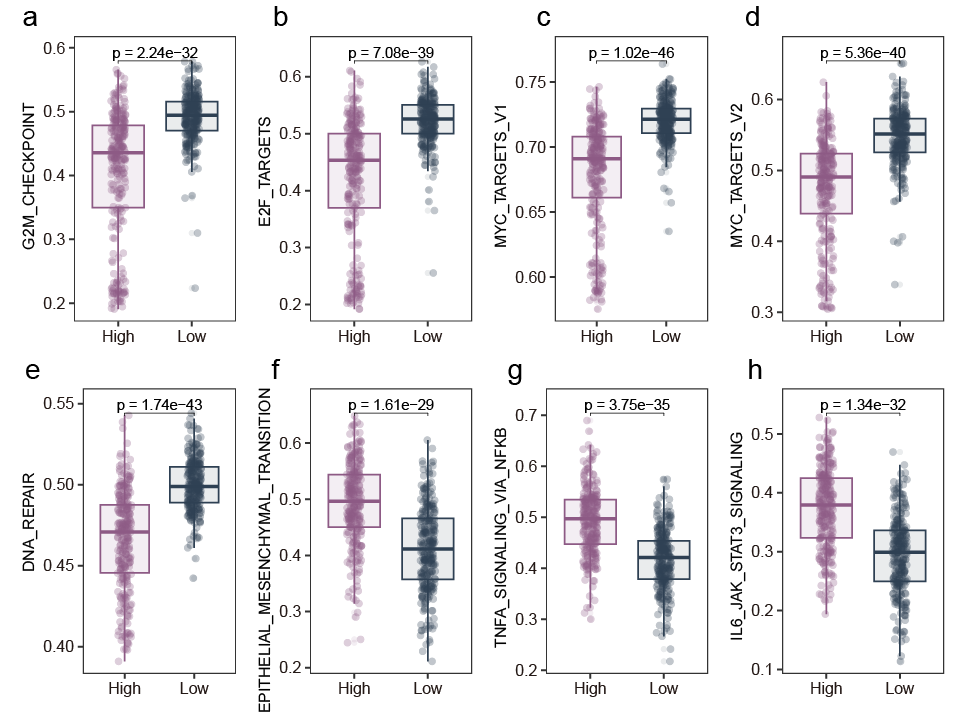


**Supplementary Figure 10. Differences in the GSVA score of cancer hallmarkers between the high- and low-risk group.** (a) G2M checkpoint. (b) E2F targets. (c) MYC targets v1. (d) MYC targets v2. (e) DNA repair. (f) EMT. (g) TNFA signaling via NFkB. (h) IL6-JAK-STAT3. Wilcoxon test was used to generate the *P* values.


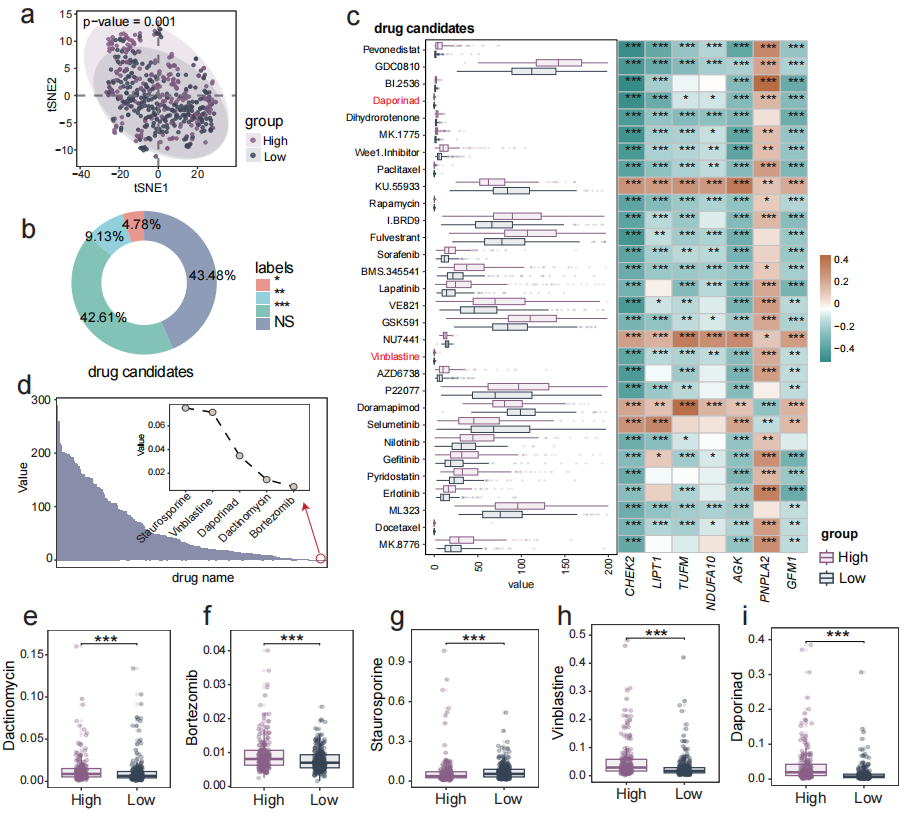


**Supplementary Figure 11. Drug sensitivity analyses in the high- and low-risk groups.** (a) Difference in the IC50 value of 198 drugs between the high- and low-risk groups. (b) Statistical significance of the differences of the IC50 value of the drugs between the two groups. (c) Boxplots on the left showing the top 30 drugs with significant difference between the two groups. Heatmap on the right showing the correlation between the LM.Sig and the 30 drugs. (d) Barplot showing the average IC50 value of all the drugs. The built-in graph showing the five drugs with the lowest IC50 values. (e-i) Boxplots showing the difference in the IC50 value of the five drugs between the two groups. Wilcoxon test was used to generate the *P* value. * *P* < 0.05, ** *P* < 0.01, *** *P* < 0.001.

**Supplementary Table 1. Detailed information of 11 ICI cohorts.**

| Cancer Type | Source | Dataset ID | NR | R | Total |
| --- | --- | --- | --- | --- | --- |
| NSCLC | GEO:GSE126044 | Cho_NSCLC_aPD1 | 11 | 5 | 16 |
|  | GEO:GSE135222 | Kim_NSCLC_aPD1 | 19 | 8 | 27 |
|  | GEO:GSE166449 | Lee_NSCLC_aPD1 | 15 | 7 | 22 |
| SKCM | PMID: 31792460 | Liu_SKCM_aPD1 | 72 | 49 | 121 |
|  | GEO:GSE78220 | Hugo_SKCM_aPD1 | 13 | 15 | 28 |
|  | PMID: 26359337 | Allen_SKCM_aCTLA4 | 90 | 63 | 153 |
| BLCA | GEO:GSE176307 | Kim_BLCA_aPD1 | 72 | 16 | 88 |
|  | IMvigor210 | IMvigor210_BLCA_aPD1 | 230 | 68 | 298 |
| GBM | PMID: 30996326 | Zhao_GBM_aPD1 | 17 | 17 | 34 |
| RCC | PMID: 32472114 | Braun_RCC_aPD1 | 133 | 39 | 172 |
| STAD | ENA:PRJEB25780 | Kim_STAD_aPD1 | 57 | 21 | 78 |
| Total | | | 729 | 308 | 1037 |

**Supplementary Table 2. Ten previously established ICI response models.**

| Signature name | PMID |  |
| --- | --- | --- |
| IFNG | 28650338 | IDO1、CXCL10、CXCL9、HLA-DRA、STAT1、IFNG |
| T cell inflamed GEP | 28650338 | CD3D、IDO1、CIITA、CD3E、CCL5、GZMK、CD2、HLA-DRA、CXCL13、IL2RG、NKG7、HLA-E、CXCR6、LAG3、TAGAP、CXCL10、STAT1、GZMB |
| NLRP3 inflammasome | 33212483 | ARRDC1-AS1、CARD8、GSDMD、ATAT1、CD36、CPTP、DHX33、EIF2AK2、GBP5、NLRC3、PYDC2、SIRT2、TLR4、TLR6、USP50、APP、CASP1、HSP90AB1、MEFV、NFKB1、NFKB2、NLRP3、P2RX7、PANX1、PSTPIP1、PYCARD、RELA、SUGT1、TXN、TXNIP |
| Cytotoxic | 25594174 | HLA-A、HLA-B、HLA-C、CASP8 |
| ImmuneCells | 33033253 | ADAM21、ALDH1L2、APBB2、APOC2、ARSF、ASAH2、ASPM、BIRC5、C6orf223、CACNG1、CCL18、CD177、CD244、CDCA5、CDH1、CEACAM6、CILP2、CLDN7、CLNK、CORO7、CR2、CRTAM、CYTL1、DHRS9、DLK1、DUSP13、EIF4A2、ENTHD1、FBLN1、FBLN2、FMOD、FOLR2、FOXI1、GBP1P1、GDF1、GIMAP4、GPR31、GRIA1、GRM7、ITGA3、JMJD7、KIR2DL4、KIRREL2、KLHDC8B、KRT4、LALBA、LEF1、LINC00243、LYSMD2、MAEL、MAP2K5、MATN3、MFAP2、MKI67、MMP12、MMP9、MT1G、MUSK、MXRA8、MYL1、MYO1G、NACA2、NACA3P、NUDT10、NUPR1、OTOF、PCLAF、PKDCC、PLA2G2D、PPA2、PPP4R3C、PRPH、PRUNE2、RASL12、RIMS2、RNASE1、ROR1、RPL36AP41、RRM2、SCGB2A2、SELENOP、SH2D2A、SHC3、SLC16A3、SPATA13、SPC24、SPP1、STC1、STC2、STOML3、SYT6、TDRD15、TEAD2、TK1、TM4SF19、TMEM171、TRAF3IP2、TREM2、TRPC4、TSHZ3、TUBA8、TYMS、UBE2C、UNC80、ZNF219、ZNF462、ZNF610、ZNF880 |
| T Cell Exclusion | 30388455 | AHCY、APP、ATP5D、ATP5G3、BOP1、BTF3、BZW2、C17orf76-AS1、C19orf48、C1QBP、C6orf48、CACYBP、CCT3、CCT4、CCT6A、CCT7、CDCA7、CDK4、CHCHD2、CTPS1、DARS、DCTPP1、DDX21、EEF1B2、EEF1D、EEF1G、EEF2、EIF2S3、EIF3E、EIF3F、EIF3G、EIF3K、EIF3M、EIF4A1、ENO1、EXOSC5、FAM92A1、FARSA、FBL、FKBP4、GAS5、GGH、GNB2L1、GNL3、GPATCH4、GPI、HMGB1、HNRNPA1、HNRNPC、HNRNPH1、HNRNPM、HSPD1、IDH2、IFRD2、ILF2、ILF3、IMPDH2、ISYNA1、LDHB、LSM4、LSM7、LYPLA1、MAGEC1、MCM7、MDH2、MKI67IP、MRPL15、MRPL37、MRPL4、MRPS12、NACA、NCL、NDUFA11、NME1、NME2、NOLC1、NOP16、NPM1、NREP、PABPC1、PAICS、PFN1、PLEKHJ1、POLD2、POLR1D、POLR2E、PPA1、PPIA、PRMT1、PTMA、PUF60、RPL10、RPL10A、RPL11、RPL12、RPL13、RPL13A、RPL13AP5、RPL14、RPL15、RPL17、RPL18、RPL18A、RPL19、RPL21、RPL22、RPL26、RPL27、RPL27A、RPL28、RPL29、RPL3、RPL30、RPL31、RPL32、RPL35、RPL36、RPL36A、RPL37、RPL37A、RPL39、RPL4、RPL41、RPL5、RPL6、RPL7、RPL7A、RPL8、RPL9、RPLP0、RPLP1、RPLP2、RPS10、RPS11、RPS13、RPS14、RPS15、RPS15A、RPS16、RPS17、RPS17L、RPS18、RPS19、RPS2、RPS20、RPS21、RPS23、RPS24、RPS25、RPS27、RPS27A、RPS28、RPS3、RPS3A、RPS4X、RPS5、RPS6、RPS7、RPS8、RPS9、RPSA、RQCD1、RRS1、RSL1D1、RUVBL2、SAE1、SERBP1、SERPINF1、SET、SHMT2、SLC19A1、SLC25A13、SLC25A6、SMARCA4、SMIM15、SNHG15、SNHG6、SNRPB、SNRPC、SNRPD1、SNRPD2、SNRPE、SOX4、SRM、SSB、SSR2、TIMM13、TIMM44、TIMM50、TOP1MT、TPI1、TRAP1、TRIM28、TUBB、TYMS、UBA52、UCK2、UHRF1、UQCRFS1、UQCRH、VDAC2、XIST、ZFAS1 |
| CRMA | 29656892 | MAGEA3、CSAG3、CSAG2、MAGEA2、MAGEA2B、CSAG1、MAGEA12、MAGEA6 |
| IMPRES | 30127394 | PD-1、CD27、CTLA4、CD40、CD86、CD28、CD80、PDL-1、HVEM、OX40L、CD137L、VISTA、TIM-3、CD200、CD276 |
| IPRES | 31683225 | ANGPT2、AXL、CCL13、CCL2、CCL7、CDH1、FAP、FLT1、1L10、LOXL2、RORS、TAGLN、TWIST2、VEGFC、VEGFA、WNT5A |
| TRS | 34804045 | CTLA4、CXCR6、LYST、CD38、GBP2、HLA-DRB5 |
| IMS | 33542239 | FAP、PDGFRB、CD163、SIGLEC1、IL10、CCL2、CCL8、CCL13、INHBA、VCAN、AXL、TWIST2、ADAM12、COL6A3、STC1、ISG15、BCAT1、OLFML2B |

**Supplementary Table 3. The correlation between the LM.Sig and the TME characteristics.**

| LM-related gene | ESTIMATE | Correlation | pvalue |
| --- | --- | --- | --- |
| CHEK2 | StromalScore | -0.4608 | 0.000 |
| LIPT1 | StromalScore | -0.2173 | 0.000 |
| TUFM | StromalScore | -0.3712 | 0.000 |
| NDUFA10 | StromalScore | -0.3844 | 0.000 |
| AGK | StromalScore | -0.3920 | 0.000 |
| PNPLA2 | StromalScore | 0.0807 | 0.072 |
| GFM1 | StromalScore | -0.3517 | 0.000 |
| CHEK2 | ImmuneScore | -0.3787 | 0.000 |
| LIPT1 | ImmuneScore | -0.0236 | 0.599 |
| TUFM | ImmuneScore | -0.2550 | 0.000 |
| NDUFA10 | ImmuneScore | -0.2062 | 0.000 |
| AGK | ImmuneScore | -0.2905 | 0.000 |
| PNPLA2 | ImmuneScore | 0.1825 | 0.000 |
| GFM1 | ImmuneScore | -0.3497 | 0.000 |
| CHEK2 | Tumourpurity | 0.4414 | 0.000 |
| LIPT1 | Tumourpurity | 0.1161 | 0.009 |
| TUFM | Tumourpurity | 0.3288 | 0.000 |
| NDUFA10 | Tumourpurity | 0.3086 | 0.000 |
| AGK | Tumourpurity | 0.3546 | 0.000 |
| PNPLA2 | Tumourpurity | -0.1361 | 0.002 |
| GFM1 | Tumourpurity | 0.3732 | 0.000 |

**Supplementary Table 4. Baseline characteristics of 42 advanced NSCLC patients.**

| Baseline characteristics of 42 advanced NSCLC patients | | |
| --- | --- | --- |
| Sample number | N=42 | |
| Mean age | 57.8 (35-77) | |
| Gender | Male | 33 (78.6) |
|  | Female | 9 (21.4) |
| Smoke status | Smoker | 21 (50) |
|  | Former smoker | 3 (7.1) |
|  | Non-smoker | 18 (42.9) |
| Histology subtype | Adenocarcinoma | 18 (42.9) |
|  | Squamous carcinoma | 18 (42.9) |
|  | Other | 6 (14.3) |
| Stage | III b/c | 14 (33.3) |
|  | IV | 28 (66.7) |
| Driver genes | EGFR | 9 (21.4) |
|  | ALK | 2 (4.8) |
|  | RET | 1 (2.4) |
|  | HER2 | 2 (4.8) |
|  | Non-driver | 24 (57.1) |
|  | Unknown | 4 (9.5) |
| Time of biopsy | Before systematic treatment | 35 (83.3) |
|  | After failure to TKI | 2 (4.8) |
|  | After failure to immunotherapy | 3 (7.1) |
|  | After failure to chemotherapy | 2 (4.8) |

**Supplementary Table 5. Mediation linkages for the impact of the genus *Lachnoclostridium* on immune cells through the LM.Sig**

| genus | LM.Sig | cell | Prop.  mediated | ACMEp |
| --- | --- | --- | --- | --- |
| *Lachnoclostridium* | CHEK2 | CD4+ T cells | 0.208 | 0 |
| *Lachnoclostridium* | TUFM | CD4+ T cells | 0.091 | 0.004 |
| *Lachnoclostridium* | PNPLA2 | CD4+ T cells | 0.306 | 0.036 |
| *Lachnoclostridium* | GFM1 | CD4+ T cells | 0.392 | 0 |
| *Lachnoclostridium* | CHEK2 | CD8+ T cells | 0.104 | 0 |
| *Lachnoclostridium* | TUFM | CD8+ T cells | 0.062 | 0 |
| *Lachnoclostridium* | NDUFA10 | CD8+ T cells | 0.056 | 0 |
| *Lachnoclostridium* | AGK | CD8+ T cells | 0.065 | 0.004 |
| *Lachnoclostridium* | GFM1 | CD8+ T cells | 0.166 | 0 |
| *Lachnoclostridium* | GFM1 | NK cells | 0.081 | 0.008 |
| *Lachnoclostridium* | CHEK2 | Myeloid dendritic cells | 0.145 | 0 |
| *Lachnoclostridium* | TUFM | Myeloid dendritic cells | 0.105 | 0 |
| *Lachnoclostridium* | NDUFA10 | Myeloid dendritic cells | 0.068 | 0 |
| *Lachnoclostridium* | AGK | Myeloid dendritic cells | 0.049 | 0.004 |
| *Lachnoclostridium* | GFM1 | Myeloid dendritic cells | 0.151 | 0 |
